# Cell-specific models of hiPSC-CMs developed by the gradient-based parameter optimization method fitting two different action potential waveforms

**DOI:** 10.1038/s41598-024-63413-0

**Published:** 2024-06-07

**Authors:** Yixin Zhang, Futoshi Toyoda, Yukiko Himeno, Akinori Noma, Akira Amano

**Affiliations:** 1 Graduate School of Life Sciences, Ritsumeikan University, Kusatsu, Japan; 2 Department of Physiology, Shiga University of Medical Science, Otsu, Japan; 3 Central Research Laboratory, Shiga University of Medical Science, Otsu, Japan

**Keywords:** Cardiovascular biology, Computational models

## Abstract

Parameter optimization (PO) methods to determine the ionic current composition of experimental cardiac action potential (AP) waveform have been developed using a computer model of cardiac membrane excitation. However, it was suggested that fitting a single AP record in the PO method was not always successful in providing a unique answer because of a shortage of information. We found that the PO method worked perfectly if the PO method was applied to a pair of a control AP and a model output AP in which a single ionic current out of six current species, such as *I*_Kr_, *I*_CaL_, *I*_Na_, *I*_Ks_, *I*_Kur_ or *I*_bNSC_ was partially blocked in silico. When the target was replaced by a pair of experimental control and *I*_Kr_-blocked records of APs generated spontaneously in human induced pluripotent stem cell-derived cardiomyocytes (hiPSC-CMs), the simultaneous fitting of the two waveforms by the PO method was hampered to some extent by the irregular slow fluctuations in the *V*_m_ recording and/or sporadic alteration in AP configurations in the hiPSC-CMs. This technical problem was largely removed by selecting stable segments of the records for the PO method. Moreover, the PO method was made fail-proof by running iteratively in identifying the optimized parameter set to reconstruct both the control and the *I*_Kr_-blocked AP waveforms. In the lead potential analysis, the quantitative ionic mechanisms deduced from the optimized parameter set were totally consistent with the qualitative view of ionic mechanisms of AP so far described in physiological literature.

## Introduction

The human cardiac action potential (AP) is characterized by the long-lasting plateau phase of the action potential, which lasts for a few hundreds of milliseconds. The AP plateau phase is generated by several ionic currents such as *I*_CaL_, *I*_NaL_, *I*_Kr_, *I*_Ks_, *I*_Kur_, and *I*_bNSC_, whose conductances are relatively small to allow subtle adjustment of the AP configuration as well as the force of contraction through a variety of cellular control mechanisms. The property of individual ionic currents has been extensively studied for more than a half century, and findings were integrated into computer models to reconstruct not only the membrane excitation but also the intracellular Ca^2+^ dynamics and the force of contraction of cardiac myocytes^1–6^. The models of human cardiac myocytes^7–10^ are used to examine the physiological mechanisms, pharmacological regulation, and pathophysiological alterations of these cellular functions. Thus, computer models of cardiac myocytes are now becoming an essential facility in the laboratory.

To extend the usage of the computer model in predicting responses of cardiac myocytes to various kinds of experimental interventions, the accuracy of the model should be assessed in an objective manner, as has been done in various biological mechanisms^11–19^. The validation of the computer model of cardiac myocytes so far described in literature relies upon statistical comparison between the model output and the experimental recordings with respect to the AP metrics, such as the AP duration (APD), the level of the AP plateau as well as the Ca^2+^ transients at different stimulus frequency. However, quantitative parameter optimization (PO) of the computer model is required in reference to experimental measurements (see Whittaker et al.^13^ for the recent review article).

Dokos and Lovell^11^ firstly applied the gradient-based parameter optimization method to the computer model of cardiac membrane excitation^20^. Model parameters such as the conductance of the four ionic currents and the rate constants of state transition of the four ionic currents (63 parameters in total) were optimized. They examined the accuracy of the PO method by testing if the original AP waveform (model output) can be recovered from the modified AP waveform, both of which were generated by the computer model. (We call this mode of PO method as model vs model (MM) test in comparison to the experimental data vs model (EM) test using the experimental recordings in the present study.) They concluded, however, that it was difficult to recover the original model parameters. They suggested the feasibility of the PO method will be improved if provided with additional experimental data.

Recently, Groenendaal et al.^21^ applied the MM test to a limited number of conductance parameters of the detailed human cardiac myocyte model^8^. They used the PO method based on the algorithm of genetic evolution^22^. In addition to the AP waveform, they used a current response, which was generated by applying voltage clamp pulses to the cardiac cell model. For the error function, the AP metrics were used. Thus, they could obtain a parameter set which well superimposed the model output to the target AP waveform. However, it has been suggested that different combinations of parameters may generate simple outputs that are very similar^11,21,23,24^. We applied the gradient-based PO method to variable AP configurations of the hiPSC-CMs^25^. It was found that the model parameters were slightly variable by less than 5% of the mean in each cell model when the mean square error (MSE) was finally minimized by the PO method. However, significant positive or negative correlations were observed between different pairs of parameters which could be attributed to the overlapping of the two currents during the slow diastolic depolarization (SDD) or the AP plateau. Thus, different combinations of two parameters obtained from the correlation curve can generate similar AP waveforms. This complication might be solved by using additional information obtained by different experimental protocols. Namely, a unique parameter set will be determined by the gradient-based PO method at a cross point of correlation lines or curves within a multi-dimensional parameter space.

In the present study, we used two experimental records of spontaneous AP obtained before and during partial blockage of *I*_Kr_ for the PO method. The baseline model of hiPSC-CMs developed by Kohjitani et al.^25^ was used to obtain individual cell-specific models.

## Methods

### Preparation of dissociated hiPSC-CMs and recording of spontaneous APs

The preparation of dissociated hiPSC-CMs and the electrophysiological recording of APs were similar to those described in Kohjitani et al.^25^ The cells were purchased from Myoridge, Kyoto, Japan (CarmyA, H-011106). The frozen single cell stocks were rapidly thawed, suspended in CarmyA seeding medium (ME-12A00241, Myoridge) supplemented with 10 μM Rock inhibitor, Y-27632 (Sigma), and then sparsely plated on glass coverslips precoated with 1.6 µg/ml iMatrix-511 silk (Matrixome, Osaka, Japan). After 10–21 days of culture in CarmyA maintenance medium (ME-01A00241, Myoridge), spontaneously beating single cells were used for the experiments. For the electrophysiological measurements, the cells attached on the glass coverslip were transferred into the recording chamber mounted on the microscope stage and superfused with Tyrode solution containing (in mM): 140 NaCl, 0.33 NaH_2_PO_4_, 5.4 KCl, 1.8 CaCl_2_, 0.5 MgCl_2_, 5 HEPES, 5.5 glucose; pH7.4 (NaOH) at 37 °C. APs were recorded in the amphotericin-perforated patch-clamp configuration under current-clamp mode using an EPC8 patch-clamp amplifier in combination with PatchMaster acquisition software (HEKA). Pipette solution contained (in mM): 70 KOH, 50 KCl, 10 KH_2_PO_4_, 1 MgSO_4_, 3 Na_2_-ATP, 5 HEPES, 5 EGTA, 0.1 Li_2_-GTP; pH 7.2 (aspartate). The AP signal was filtered at 5 kHz and digitized at 20 kHz. A selective *I*_Kr_ blocker, E-4031 (Wako, Japan) was dissolved in distilled water to yield 5 mM stock solution and added to the bath solution at the concentrations of 0.01–1 μM.

### The baseline model of hiPSC-CM membrane excitation

The baseline model of hiPSC-CMs is a variant of the human ventricular cell model (hVC model) adapted to experimental measurements in hiPSC-CMs^25^. The hVC model has been fully described in literature^9,10^ and shares many comparable characteristics with other human models so far published^7,8^. The model structure of the hVC model consists of the cell membrane with fifteen ionic channel species and two ion exchangers, the sarcoplasmic reticulum equipped with the refined Ca^2+^ releasing units coupled with the L-type Ca^2+^ channels on the cell membrane at the microscopic dyadic space, the contractile fibers, and the cytosolic Ca^2+^ diffusion spaces containing several Ca^2+^-binding proteins.

The source code of the present hiPSC-CM model was written in Visual Basic and is available from our e-Heart website (http://www.eheartsim.com/en/downloads/), and the model equations are described in the supplemental materials in the present study.

In the present study, the net membrane ion current (*I*_tot_cell_) is calculated as the sum of nine ion channel currents and two ion transporters (*I*_NaK_ and *I*_NCX_) (Eq. 1).

1 $$\begin{array}{c}{I}_{tot\_cell}={I}_{Na}+{I}_{CaL}+{I}_{ha}+{I}_{K1}+{I}_{Kr}+{I}_{Ks}+{I}_{Kur}+{I}_{Kto}+{I}_{bNSC}+{I}_{NaK}+{I}_{NCX} \end{array}$$

A few ion channels listed in Eq. (1) or in supplemental materials were not necessarily expressed in every cell to a significant level to affect the AP waveform. In such cases, we removed the current from the initial set of PO parameters through the initial manual fitting process explained in the last part of this section.

The *I*_Na_ is composed of two components; one is the transient type (*I*_NaT_) and the other shows the very slow inactivation (*I*_NaL_). *I*_NaL_ plays a significant role in both slow diastolic depolarization as well as in the AP plateau. In the present study, the Na^+^ mediated sustained inward current, *I*_st_^26–28^ is included in the *I*_CaL_ for simplicity. Rationale for this simplification was derived from the references suggesting Cav1.3 as a molecular determinant of *I*_st_^29,30^. We excluded background currents of much smaller amplitude, such as *I*_KACh_*, I*_KATP_*, I*_(lCa)_, and *I*_Cab_ from the parameter optimization and adjusted only the background non-selective cation current (*I*_bNSC_) of significant amplitude for the sake of simplicity^31–33^. The *I*_bNSC_ was re-defined in the present study as a time-independent net current, which remained after blocking all time-dependent currents.

The parameter optimization in the present study was conducted under the assumption that the variable AP configurations in the hiPSC-CMs were caused by different expression levels of ion channels on the cell surface membrane, while the channel open- and close-state transition kinetics remain the same as described in the hVC cell model. Before starting the PO method, we manually adjusted the model parameters (current magnitude of individual currents in Eq. 1) to fit the model output to the target AP waveform on the graphic display. Usually, the 9 ionic channel currents were all examined for their capability to simulate the experimental AP configuration by the model output, and those without an obvious improvement of the model output were excluded from the PO parameters. This selection of the optimizing parameters contributes to reducing the computational time of the PO method. The computational time increases exponentially when the number of the optimizing parameters increase and also if the parameter which does not affect the AP waveform was included. Note that, the number of the optimizing parameters to perform the PO method in the realistic computational time was around six with the computational resource used in this study.

### A brief note on membrane excitation

The membrane excitation of the model is generated through charging and discharging the membrane capacitance (*C*_*m*_) by the net ionic current (*I*_tot_cell_) across the cell membrane (Eq. 2). The driving force for the ionic current is provided by the potential difference between *V*_m_ and the equilibrium potential (*E*_x_) (Eq. 3). The net electrical conductance of the channel is changed by the dynamic changes in the open probability (*pO*) of the channel, which is mostly *V*_m_-dependent through the *V*_m_-dependent rate constants ($\alpha , \beta$) of the opening and closing conformation changes of the channel (Eqs. 4, 5).

2 $$\frac{{dV}_{m}}{dt}=-\frac{\sum {I}_{tot\_cell}}{{C}_{m}}$$

3 $${I}_{x}={\overline{G} }_{x}\cdot pO\cdot \left({V}_{m}-{E}_{x}\right)$$

4 $$\frac{dpO}{dt}=\alpha \cdot \left(1-pO\right)-\beta \cdot pO$$

5 $${\left[\alpha \beta \right]}^{T}=f\left({V}_{m}\right)$$

For the parameter optimization, the limiting conductance ${\overline{G} }_{x}$ of each ionic current (Eq. 3) is represented by a product of *I*_xmag_ and *I*_xsf_. The former is a reference magnitude of conductance and the latter is the scaling factor (0 < *I*_xsf_).

6 $$\overline{G}_{x} = I_{x,mag} \cdot {\text{I}}_{xsf}$$

When the constant field (GHK) equation is used, the current density amplitude *I*_x_ is given by,

7 $$I_{x} = \overline{P}_{x} \frac{{z \cdot F \cdot A \cdot \left( {S_{i} - S_{o} \cdot B} \right)}}{1 - B} \cdot pO$$

8 $$A= \frac{z\cdot F\cdot {V}_{m}}{R\cdot T}$$

9 $$B={e}^{-A}$$

where ${\overline{P} }_{x}$ is the limiting permeability of ion x across the cell membrane and *S*_i_ and *S*_o_ are the internal and external ion concentrations, respectively. The unit of ${\overline{P} }_{x}$ is pA/pF/mM across the membrane. The *I*_xsf_ is also used for the PO parameter.

10 $$\overline{P}_{x} = P_{x,mag} \cdot {\text{I}}_{xsf}$$

### Pattern search method; MM test, EM test, and multi-run test

For a system showing a relatively simple gradient of MSE along the parameter axis, gradient-based optimization methods are more efficient in general than stochastic methods. We used one of the basic gradient-based optimization methods, the pattern search (PS) method^34–36^. The computer program code of the pattern search^37^ is simple and does not require derivatives of the objective function. We implemented the program code into a homemade program for data analysis.

The parameter optimization is driven by calculating the error function which uses the mean square error (MSE) between the target AP waveform (*V*_m,t_) and the output of the baseline model (*V*_m,a_) (Eq. 11),

11 $$MSE=\frac{\sum {\left({V}_{m,a}-{V}_{m,t}\right)}^{2}}{N}$$

where *V*_m,a_ represents adaptive *V*_m_ (the model output) generated by adjusting the randomized set of *I*_xsf_s in the baseline model. The target *V*_m,t_ is the AP waveform of the intact baseline model. The magnitude of MSE was stabilized by obtaining a stable rhythm of spontaneous APs through repetitive numerical integration of the model, usually, 30 ~ 100 spontaneous cycles were repeated for a new set of *I*_xsf_s. The MSE was calculated within a time window which included a single cycle of the spontaneous AP generation. N is the number of digitized *V*_m_ points with a time interval of 0.1 ms.

The present study used the weighted sum of MSE. The single cycle of the spontaneous AP was separated into three segments. The first segment starts from − 20 mV during the repolarizing phase to maximum diastolic potential (MDP) and the weight is 5 ~ 6. The second segment covers MDP, SDD, and the foot of AP discharge and the weight is 1, and the third segment covers the AP plateau until to repolarizing phase to − 20 mV with weight = 0.3 or 0.5. In the first segment, the magnitude of *I*_Kr_ dominates to allow its determination by the PO method. The second segment is influenced mostly of *I*_bNSC_, *I*_ha_ in addition to *I*_NaL_ and *I*_CaL_, which takes a major role in generating the exponential depolarization in the late half of SDD. The third segment consists of the AP plateau, generated by *I*_CaL_, *I*_NCX_, *I*_NaL_, *I*_Ks_, and *I*_Kur_. By providing large weight to the first time window, *I*_Krsf_ rapidly gets close to the optimal value independent of other parameters which means that the succeeding optimization process behaves as if the number of the optimizing parameters is one less. According to our experience, the preliminary manual fitting can be carried out smoothly in a sequence of these three segments.

All hiPSC-CMs used in the present study showed spontaneous discharge of AP. Therefore, to calculate MSE, it was essential to start all records from a particular *V*_m_ value; − 20 mV was used in this study. We calculated the model until digitized *V*_m_ crossed a range of − 20.000 mV > *V*_m_ > − 20.004 mV. Thereafter, a record of a single AP cycle of the target or model output was obtained. For the graphic display, we usually connected two cycles of AP for better visualization.

In the PS method, a base point (BP) is determined by the initial parameter set. On both sides of BP, two new points (NP) are set by a ± step to calculate MSE for each NP, and the numerical integration of the hiPSC-CM model was conducted to obtain new AP waveforms. If MSE is improved, the NP is moved by the single step in the direction giving the smaller MSE. This pattern search is repeated for all dimensions of PO parameters one by one until the decrease of MSE saturates. Then all BPs are moved to the last NPs for all PO parameters, and simultaneously the step size is decreased. The movements of BP are repeated until the step size becomes smaller than a critical step size, which was preset by the user.

The intracellular concentrations of Na^+^, Ca^2+^, and K^+^ will change every time when the parameters are varied during the PS method. To minimize the variation of the ion concentration, the activities of both *I*_NaK_ and *I*_NCX_ were regulated. For *I*_NaK_, Eq. (12) was used.

12 $${I}_{NaK}=max{I}_{NaK}\cdot {vcyc}_{NaK}\cdot {I}_{NaK, mag}\cdot {crf}_{NaK}$$

To calculate the correcting factor (*crf*_*NaK*_), Eq. (13) was used.

13 $$cr{f}_{NaK}=orgcr{f}_{NaK}-\left({stdNa}_{i}-{Na}_{i}\right)\cdot {fbGain}_{NaK}$$

where any deviation of [Na^+^] (*Na*_*i*_) from a standard [Na^+^] (*stdNa*_*i*_) was corrected by modifying *crf*_*NaK*_ used for the negative feedback regulation in reference to a constant (*orgcrf*_*NaK*_). The *fbGain*_*NaK*_ was usually set at 0.004 for a stable regulation. The *stdNa*_*i*_ = 5.8 mM and *orgcrf*_*NaK*_ of 0.2606 were used.

The same format of equation was used to regulate the *I*_NCX_ activity to keep the total of ionized and bound form of Ca (Ca content) within the cell, which is a sum of Ca content in the three Ca compartments and the sarcoplasmic reticulum (SR) ([Ca]_t_). The *fbGain*_*NCX*_ was 0.008. The *std*_*totCai*_ = 68.5 attomole and *orgcrf*_*NCX*_ = 2.4732.

### Parameter randomization and the multi-run test

The final level of MSE obtained by a single cycle of the PS method fluctuates between different cycles. Therefore, the PS cycle was repeated for 200 ~ 300 cycles (multi-run test). The MSE as well as the model parameters were given as an average of the top 20 results of smaller MSE as described in Kohjitani et al.^25^. Every run of the PS method started with a randomized set of the PO parameters obtained by Eq. (14).

14 $${I}_{xsf}=1+\left({R}_{sf}-0.5\right)\cdot af$$

*R*_*sf*_ is given by a random function and is distributed between $0\le {R}_{sf}<1$. The *af* is used to vary the amplitude of *I*_xsf_; an *af* of 0.3 is usually used to randomize *I*_xsf_ between 0.85 and 1.15. The time course of optimization was monitored by plotting *I*_xsf_ in a two-dimensional parameter space on the graphic display; log(MSE) on the ordinate against *I*_xsf_ on the abscissa.

### The simultaneous fitting of two AP waveforms in the PS method

In the present PO method, the experimental data obtained under the specific block of *I*_Kr_ using E-4031 in addition to the control AP waveform were used (see Doss et al.^38^ for the *I*_Kr_ block in hiPSC-CMs). Namely, MSE was calculated for both the control and the *I*_Kr_-blocked AP waveform with the model output, and the average of these two MSEs was used as the error function to drive the PO method.

The mechanisms underlying the modification of the cardiac AP waveform are implemented in the baseline model. Thus, the maximum diastolic potential, (MDP) as well as the slow diastolic depolarization (SDD) were shifted in the positive direction in most of hiPSC-CMs if ${\overline{G} }_{K}$ (Eq. 3) was decreased by E-4031. Through these changes in *V*_m_, the *V*_m_-dependent open-close gatings were altered according to the gating kinetics to modify the AP waveform. Furthermore, under the specific *I*_Kr_ block, a new constraint is applied to the PO; i.e. the ${\overline{G} }_{x}$ of other *I*_x_ should remain at the control magnitude. Thus, a common set of *I*_xsf_s except the *I*_Krsf_ was used for both the control and *I*_Kr_-blocked AP waveforms. Note that, this constraint was also applied in the initial manual fitting process, thus the magnitude of *I*_Kr_ block was manually estimated as the result in this process.

### The lead potential (*V*_L_) analysis

The final AP waveforms obtained by the PS method were examined for their validity by calculating the contributions of individual current components (*I*_x_s) to the AP waveform by using the lead potential (*V*_L_) analysis^9,39^. Contributions of individual I_x_s are expressed in a unit of mV instead of pA/pF,

15 $${V}_{L}= \frac{\Sigma ({G}_{x}\cdot {E}_{x})}{\Sigma ({G}_{x})}$$

16 $$\frac{{dV}_{m}}{dt}=-\frac{\left({V}_{m}-{V}_{L}\right)}{\tau }$$

17 $$\tau =\frac{{C}_{m}}{\Sigma {G}_{x}}$$

where *G*_x_ is the conductance (nS/pF) given by Eq. (3). The *V*_m_ closely followed the time course of *V*_L_ (Eq. 16) with the time constant τ (Eq. 17). *G*_x_ is the chord conductance in the case of currents described according to Ohm’s law. In the case of constant field equations, the *G*_x_ is obtained by differentiating GHK with respect to *V*_m_ (Eqs. 18 ~ 20) as the slope conductance (Eq. 19),

18 $$GHK= \frac{A\cdot \left({S}_{i}-{S}_{o}\cdot B\right)}{1-B}$$

19 $$dGHK=\frac{dGHK}{d{V}_{m}}=(\frac{\left({S}_{i}-{S}_{o}\cdot A\right)}{\left(1-B\right)}+\frac{\left({S}_{o}-{S}_{i}\right)\cdot A\cdot B}{{\left(1-B\right)}^{2}})/\frac{z\cdot F}{R\cdot T}$$

20 $${G}_{x}=\dot{{P}_{x}}\cdot dGHK$$

21 $$Ex={V}_{m}- \frac{GHK}{dGHK}$$

The reversal potential (*E*_x_) at a given *V*_m_ was determined by Eq. (21).

## Results

### Accuracy of the simultaneous two AP waveform fitting examined by the MM mode of the PO method

**Figure 1:**
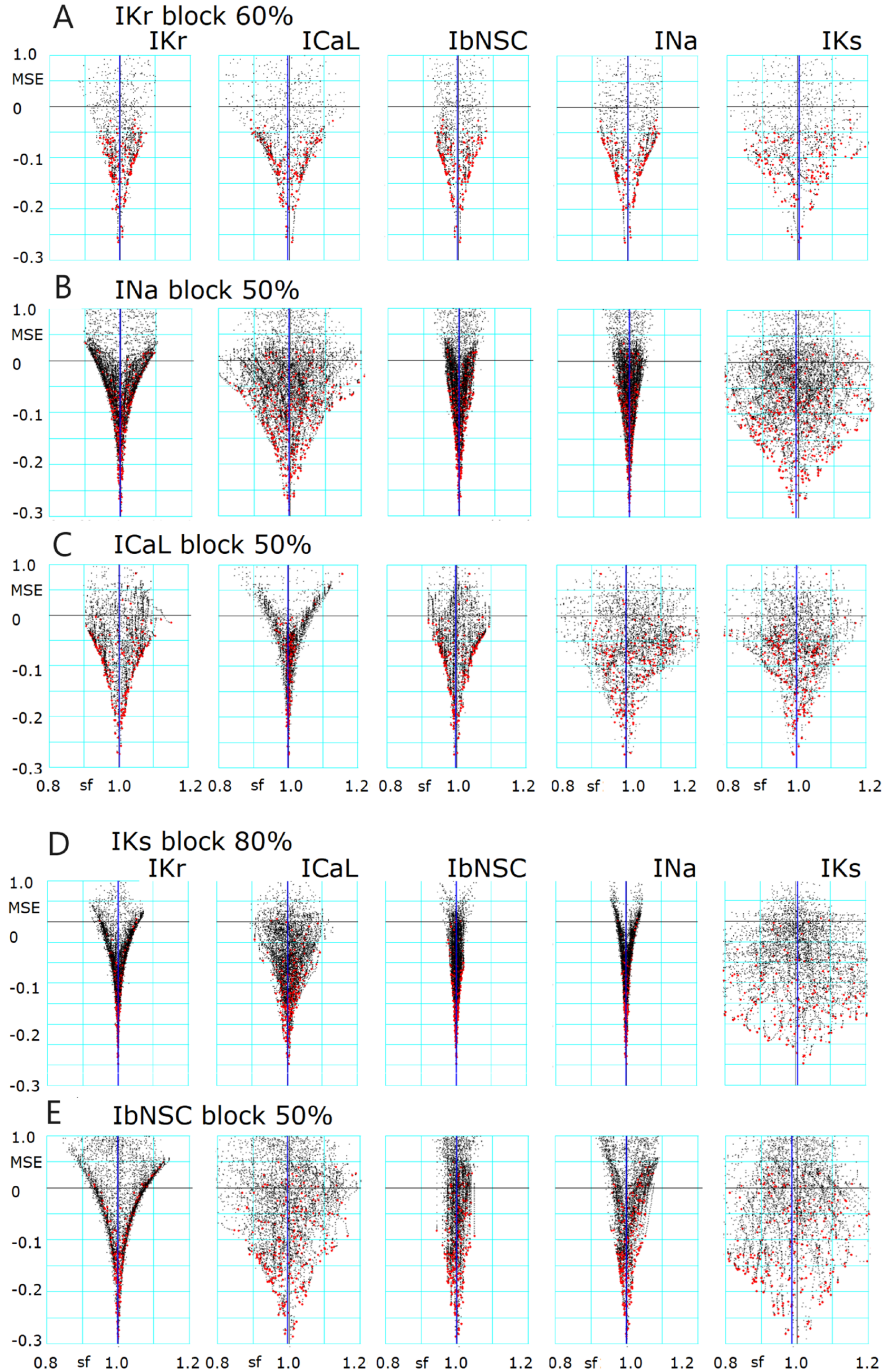
Convergence of I_xsf_ (BP) as the MSE magnitude was decreased by the simultaneous two-waveform fitting in the MM mode multi-run test. The BPs were plotted in the coordinate of log(MSE) (ordinate) vs I_xsf_ (abscissa) during the PO process by the PS method. In each line of graphs, I_Krsf_, I_Nasf_, I_CaLsf_, I_Kssf_, and I_bNSCsf_ log(MSE) were plotted. In the case of the blocked current, the I_xsf_ was normalized in reference to the blocked level.

The graphic display of the MM multi-run tests conducted for each case of selective block of *I*_Kr_ (60%), *I*_Na_ (50%), *I*_CaL_ (50%), *I*_Ks_ (80%), and *I*_bNSC_ (50%) were demonstrated in Fig. 1, where the movements of BPs in each run of PS method were plotted in the *I*_xsf_ vs log(MSE) coordinates for each *I*_xsf_ indicated at the top. The cell-specific model developed by the single AP waveform fitting was used as the baseline model. Although the PS parameters of the initial parameter set were randomized for each run of the PS method, the BPs were distributed within an obvious envelope and well converged at a normalized *I*_xsf_ = 1 value in the baseline model. The minimum level of log(MSE) was mostly smaller than -2 (MSE < 0.01 mV^2^), which is in the same range as those obtained in single AP waveform fitting (see Fig. 4 and Table 2 in Kohjitani et al.^25^).

**Figure 2:**
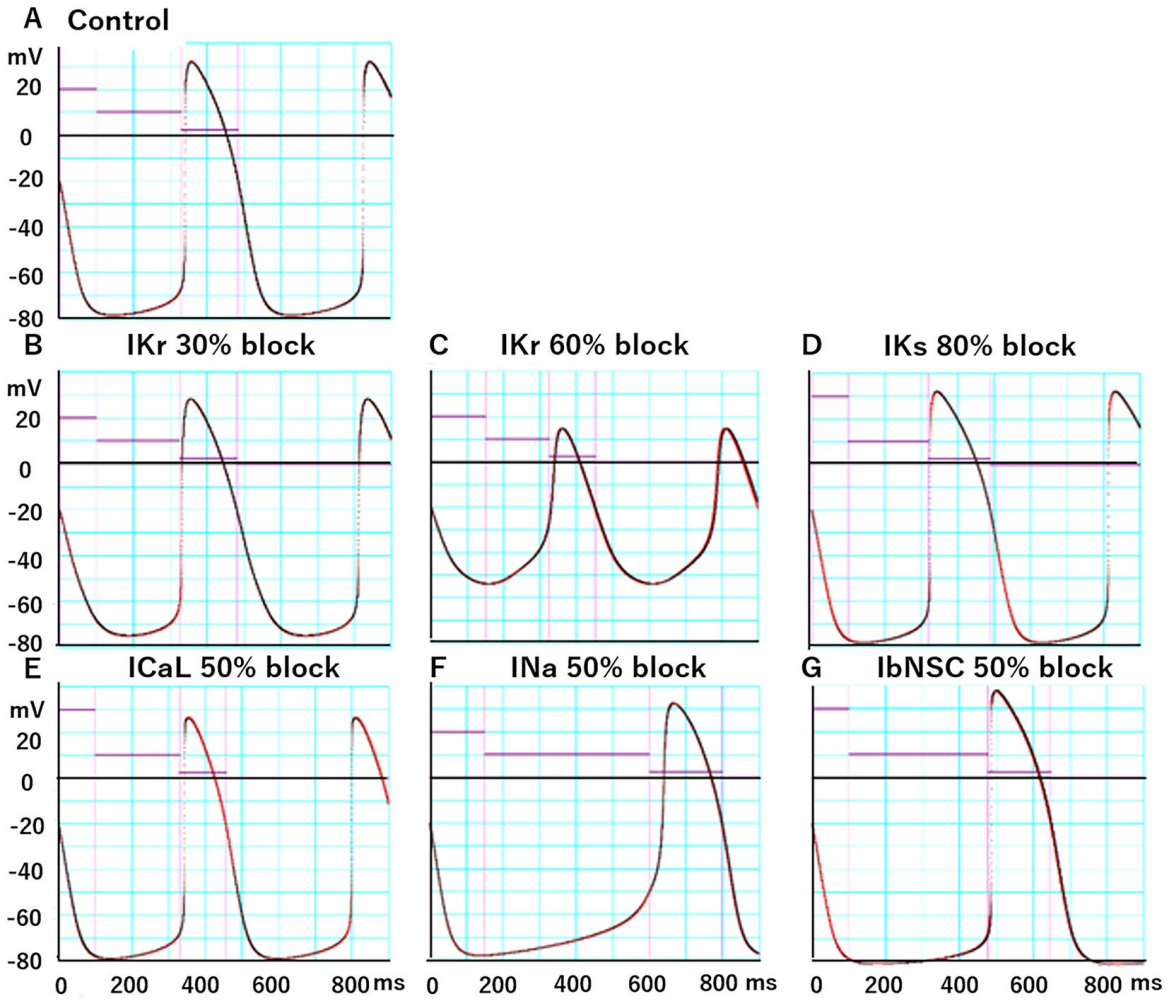
The recovery of the AP configuration from the artificial modification of AP by the PO method. The red trace was the target AP, which was generated by the intact baseline model, while the black trace was the model output generated at the end of the PO method as the result. The violet horizontal lines indicate three ranges of AP to weight (6, 1, and 0.3 for each step, independent from the ordinate scale) in calculating the weighted sum of MSE. The fitting to the control trace in (**A**) was obtained by the simultaneous two-AP-waveform fitting using the 60% I_Kr_ block. Since the fittings of the control AP in cases of any other I_x_ block were quite similar to that illustrated in (**A**), the respective control record for each I_x_ block was not demonstrated individually. Note that two different percentages of blockage (30% and 60%) were specifically tested for I_Kr_.

In Fig. 2, the target AP waveforms (red) were superimposed by APs of the model output (black traces), which were the outcomes of the MM multi-run test of the PO method in Fig. 1. It is evident that the PO was nearly perfectly conducted when the log(MSE) below − 2 were obtained. All AP waveforms well represent the characteristic effect of the *I*_x_ block. Namely, the blockage of the outward currents, *I*_Kr_ and *I*_Ks_ prolonged the APD, while the blockage of the inward currents, *I*_CaL_ shortened the APD. In the case of *I*_Na_ block, the SDD was markedly prolonged through the partial block of the *I*_NaL_ component. During the prolonged SDD, the inactivation of *I*_NaL_ might be partially removed to recover the APD. A similar prolongation of the APD was also observed by the *I*_bNSC_ blockage. These findings are totally consistent with those expected from the physiological mechanisms of the AP generation.

Table 1 lists the mean and standard deviation of the magnitude of final *I*_xsf_s in the top 20 runs of PO in the MM tests performed to generate AP waveforms demonstrated in Fig. 2. The magnitudes of *I*_xsf_ were normalized in reference to the standard values of the baseline model. In the case of blocked currents, the *I*_xsf_ was normalized to the magnitude of blockage defined in each PS method. It is evident that the error of the *I*_xsf_ is less than ~ 2% of the control and the SD of the mean was negligibly small, indicating that selective blockage of a given ionic current was correctly resolved by the PS method in the present study.

**Table 1:** List of the final I_xsf_ and the [Na^+^]_i_ and [Ca]_t_ obtained by the PS method.

TC12-MMTest	IKrsf	IKssf	IKursf	INasf	ICaLsf	Ihasf	IbNSCsf	[Na^+^]_i_	[Ca]_t_	Log(MSE)
IKr 30%	0.997	0.998		1.005	0.996	0.995	0.996	7.084	82.050	− 2.223
S.D	0.054	0.006		0.006	0.005	0.004	0.021	0.015	0.154	0.237
IKr 60%	1.017	0.995		1.004	0.998	0.985	0.995	7.015	70.430	− 2.467
S.D	0.051	0.008		0.006	0.003	0.004	0.003	0.009	0.053	0.008
IKs 80%	0.999	1.006		0.998	1.001	1.078	1.000	5.708	77.728	− 2.970
S.D	0.000	0.060		0.001	0.004	0.026	0.001	0.004	0.091	0.216
IKur 50%	1.002	0.954	1.041	0.999	1.000		1.000	6.914	87.483	− 1.916
S.D	0.003	0.057	0.070	0.012	0.013		0.007	0.030	0.002	0.196
ICaL 50%	0.977	0.995		1.003	1.001	0.999	0.998	6.596	56.277	− 2.661
S.D	0.044	0.005		0.005	0.002	0.004	0.001	0.004	0.031	0.155
INa 50%	0.989	0.999		1.000	0.998	0.988	0.997	6.916	63.736	− 2.500
S.D	0.061	0.027		0.002	0.020	0.004	0.004	0.007	0.083	0.121
IbNSC 50%	0.999	0.989	1.002	1.006	0.998		0.996	6.151	62.170	− 2.036
S.D	0.004	0.067	0.030	0.010	0.010		0.001	0.010	0.139	0.186

See the text for a more detailed explanation. The unit of [Na^+^]_i_ is mM, and that of [Ca]_t_ is attomole. [Ca]_t_ stands for the sum of ionized and bound forms of Ca (Ca_tot_) in jnc, iz, blk, and SR. Vol indicates the volume of each compartment. These concentrations were measured at the time when Vm crosses − 20 mV during the repolarization phase.

Ca_tot_ = Ca_tot,jnc_ · Vol_jnc_ + Ca_tot,iz_ · Vol_iz_ + Ca_tot,blk_ · Vol_blk_ + Ca_SRup_ · Vol_SRup_ + Ca_tot,SRrl_ · Vol_SRrl_.

The level of [Na^+^]_i_ and [Ca]_t_ indicate the final concentrations determined in the AP waveform under the selective *I*_x_ blockage. The standard deviation (SD) of the mean was also negligibly small to support that the activity of the Na/K pump current and the Na/Ca exchange currents were well controlled to give a stable level of [Na^+^]_i_ and [Ca]_t_ (Eqs. 12 and 13) in the top 20 runs of the PS method.

### Feasibility of applying the simultaneous two AP waveform fitting to experimental recordings of the selective *I*_Kr_ block

According to the MM mode fitting, it is concluded that the algorithm of the PS method is relevant to get the cell-specific model using the two-waveform parameter fitting. However, it is asked if the EM mode is also feasible in analyzing the *I*_xmag_ profile of experimental AP waveforms, which include various extra noise, such as the slow fluctuations of *V*_m_ in the hiPSC-CMs. Furthermore, the EM fitting might be interfered with if the computer model equations failed to describe the intact mechanisms of the intact cardiac myocytes. This latter problem should be finally solved by performing a new experimental protocol, but the study of the EM mode PO method might suggest inappropriate equations of the model. Here, we examine the feasibility of applying the EM mode of the PS method to the experimental AP recordings.

### Time course of the spontaneous APs after the application of E-4031 and results of the conventional PO method of using a single AP waveform

**Figure 3:**
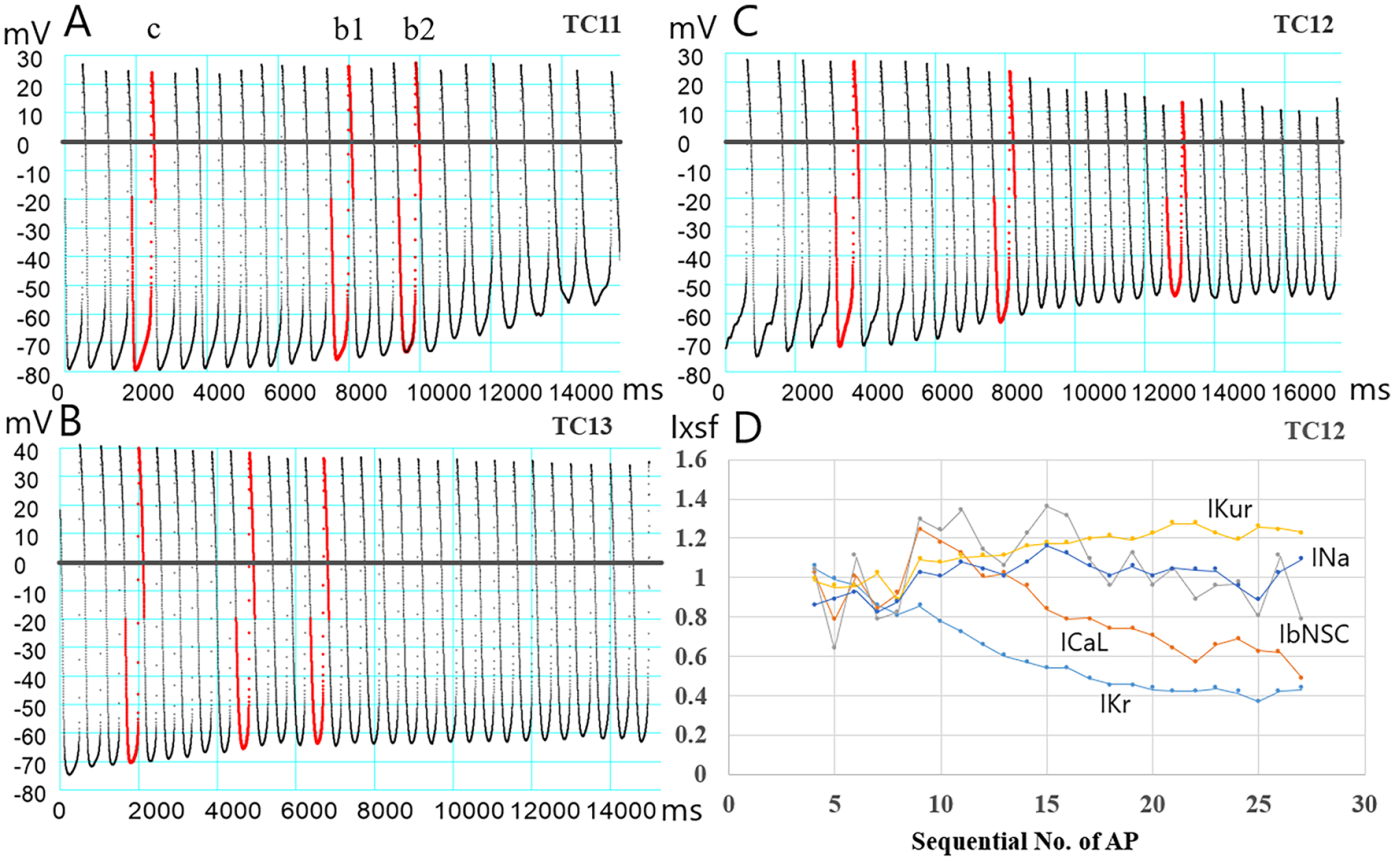
Continuous recording of the spontaneous AP generation in hiPSC-CM during the I_Kr_ block by perfusing E-4031. (**A**–**C**) Shows three examples of chart recordings of the AP waveform. The red traces indicate the single AP waveforms used for the two-waveform fitting of the PO method, which will be described in the next chapter. (**D**) Shows time courses of I_xsf_ of I_Kr_ (light blue), I_CaL_ (orange), I_Na_ (steel blue), I_Kur_ (yellow) and I_bNSC_ (gray) obtained by the conventional single waveform fitting of the PS method applied to TC12. See the text for a detailed explanation.

Three representative AP recordings in the hiPSC-CMs after exposure to an *I*_Kr_ blocker E-4031 are demonstrated in Fig. 3A–C. The continuous recordings of *V*_m_ in the hiPSC-CMs showed sporadic slow fluctuations of *V*_m_ and/or irregular cycle length of the spontaneous rhythm. In the record of a single myocyte (TC11) in Fig. 3A, for example, irregular fluctuations in MDP in addition to sporadic changes in AP configurations were obvious. The myocyte TC13 in Fig. 3B shows a relatively smooth time course of the MDP and OS change before and after the *I*_Kr_ block. In the record of myocyte TC12 in Fig. 3C, the fluctuations in AP amplitude after about 12,000 ms were of unknown origin. These fluctuations were observed in most of the hiPSC-CMs.

Before conducting the new two-waveform fitting, we firstly examined the conventional PO method to apply the baseline model to individual AP waveforms. The baseline model was obtained by applying the PS method to an AP waveform during the control condition in the same myocytes. The E-4031 was applied by switching the perfusate using a cock, which connected the outlet tube to the reservoir of test solutions about 40 cm apart from the inlet of the recording chamber to avoid mechanical vibration. It was difficult to precisely indicate the onset time of the *I*_Kr_ block by the E-4031 solution. However, the progressive positive shift of MDP induced by E-4031 was obvious. Another complication might be caused by the progress of the *I*_Kr_ block during a single AP cycle (t_2_ − t_1_). Assuming an exponential development of the *I*_Kr_ block, the progress was calculated using Eq. (22), in which *br*_t2_ indicates the block ratio at time t_2_.

22 $${{br}_{t2}= br}_{\infty }-\left({br}_{\infty }-{br}_{t1}\right)\cdot {e}^{{-\left({t}_{2}-{t}_{1}\right)}\left/ {\tau }\right.}$$

If a rate constant τ is assumed to be 30 s, the difference of *br* might be ~ 1% during the AP interval of 0.3 s. we assumed that an error due to this transitional effect might be negligibly small in testing the application of the PO method. A representative result of *I*_xsf_s optimized by the single waveform fitting of the PS method applied sequentially was illustrated for individual AP waveforms in TC12 in Fig. 3D. The *I*_Krsf_ (light blue) was decreased smoothly in an exponential manner. The fluctuations in *I*_Nasf_ (steel blue), *I*_Kursf_ (yellow) and *I*_bNSCsf_ (blue) were less than ~ 20%. However, *I*_CaLsf_ (orange)*,* largely deviated. This deviation of the *I*_CaLsf_ might be attributed not only to the extra fluctuations in the continuous recording of the spontaneous activity, but also to the shortage of information provided by the experimental single AP waveform.

### Results of applying the simultaneous two-AP-waveform fitting of the EM mode of the PS method.

**Figure 4:**
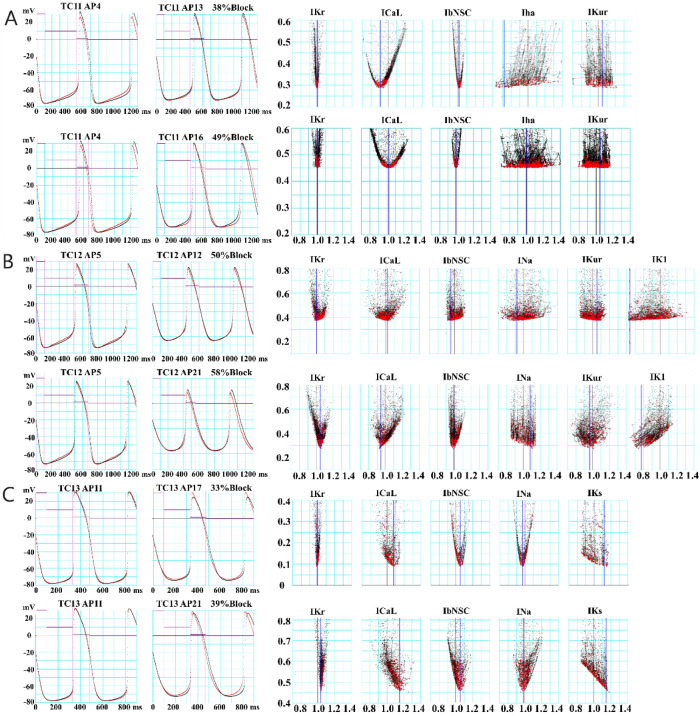
Results of the simultaneous fitting of two AP waveforms in the EM mode of the PO method. The data in three hiPSC-CMs (TC11, TC12, and TC13) were examined for the pair of control and the I_Kr_-blocked AP waveforms; the pair of c and b1, and the pair of c and b2 indicated in Fig. 3. In all panels the control APs (red) were superimposed by the model output APs (black). The results of the multi-run test are shown in the right half of figure.

From the limited length of the continuous AP record, stable AP waveforms of control and *I*_Kr_-blockage were selected for the EM mode of the simultaneous two-AP waveform fitting. The red traces in Fig. 3A–C indicated individual AP waveforms; c the reference and b1 and b2 the test waveforms used for the simultaneous fitting of pairs; c and b1, and c and b2 waveforms. Results of the PS method are shown in Fig. 4. Differently from the MM test of the PS method (Fig. 2), a slight gap remained after the end of the PO run between the target (red trace) and the model output (black trace), most probably due to the irregular slow fluctuations. Indeed, the final magnitude of log(MSE) is comparable to results obtained in the single AP wave fitting in EM mode in our previous study^25^. The result of the multi-run test shown in the right half of Fig. 4 showed clear convergence of the *I*_Krsf_, *I*_CaLsf,_ and *I*_bNSCsf_, which gave the averages of top 20 results of smaller MSEs (vertical blue lines) mostly within the ± 5% range of each standard value of 1. Note, the *I*_Krsf_ was normalized in reference to the blocked *I*_Krsf_ determined in a separate preliminary measurement. However, no obvious convergence was observed in *I*_hasf_ and *I*_Kursf_.

### Selective *I*_Kr_ block resolved by the simultaneous fitting of two waveforms of the PS method.

**Figure 5:**
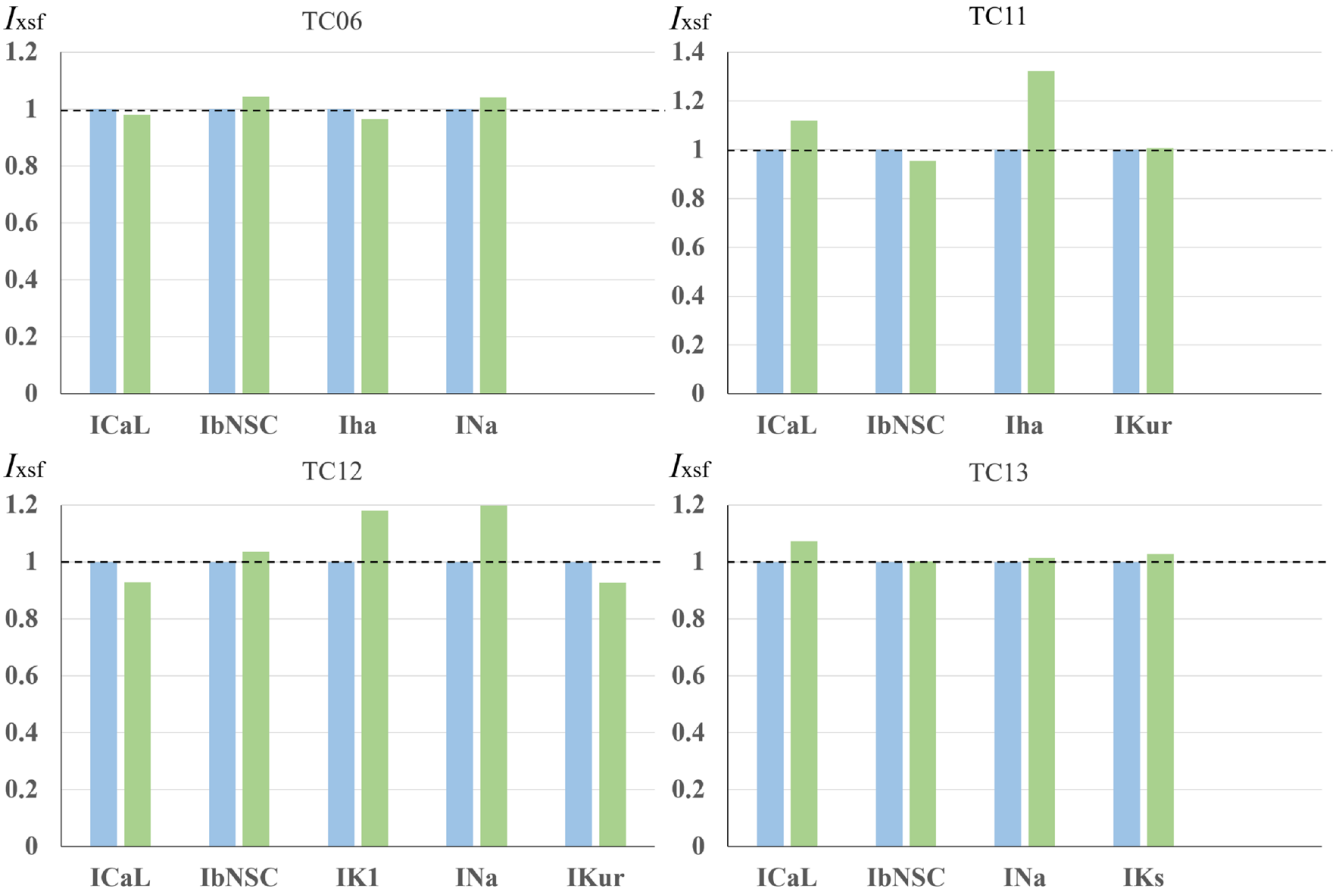
Comparison of the I_xsf_s other than I_Krsf_ examined in the EM test. The results of the two-waveform fitting using the leftmost and middle red traces shown in Fig. 3 are shown in the blue bars, and the results by using the leftmost and rightmost red traces shown in Fig. 3 are shown in the green bars. Note that the blue bars were normalized to 1.0 to compare with the green bars. See the text for the explanation.

The selective *I*_x_ block has been proved in the MM mode of the PO method (Figs. 1, 2, and Table 1). In Table 1, the *I*_xsf_ were all very close to 1, supporting the fine resolution of the selective *I*_x_ block by the PS method. In the EM mode of the PO method, however, the selective blocker which had a high selectivity to a specific channel was only available for *I*_Kr_. To prove the selective block of *I*_Kr_ obtained in the EM mode PO, the magnitudes of *I*_xsf_s other than the *I*_Krsf_ were compared in Fig. 5 at two different degrees of the *I*_Kr_ block. The magnitudes of *I*_Krsf_ were quite close to 1 in the multi-run test because the *I*_Krmag_ was adjusted to the degree of the *I*_Kr_ block, which was determined by manual fitting beforehand. For better visibility, the *I*_xsf_ obtained with the higher *I*_Kr_ block (green bars) was normalized in reference to that obtained at the lower *I*_Kr_ block (blue bars in Fig. 5). It is evident that the deviation of *I*_xsf_ was less than 10% in 16 measurements of *I*_Krsf_, and 10–20% in other 5 measurements. The large deviation of *I*_ha_ in TC11 was partly attributed to the depolarized MDP level at the higher *I*_Kr_ block, which will be discussed further in the next section. Considering the relatively large fluctuations of the experimental *V*_m_ recordings (Fig. 3), it might be concluded that the EM mode of the PS method successfully revealed the selective *I*_Kr_ block.

### Correlation between two ion currents revealed by the EM test of PS method

**Figure 6:**
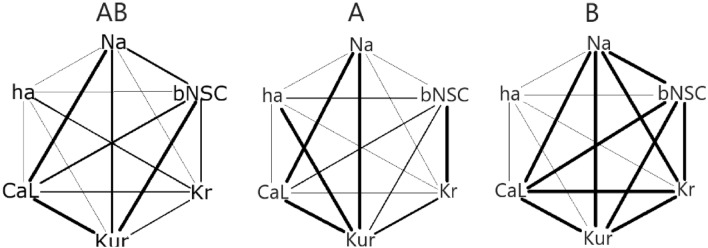
The patterns of the correlations between different pairs of I_xsf_s were compared among three different POs. The correlation of all 15 pairs of I_xsfs_ using the top 20 results obtained in the multi-run PO method for TC12 by using the leftmost and middle red traces shown in Fig. 3C (AB), by using the leftmost red trace only (**A**), and by using the middle red trace only (**B**) are shown with the lines of different thickness. The thicker lines indicate R^2^ higher than 0.8, 0.6 and 0.4. See text for more details.

When the error function MSE of two parameter sets are decreased to a minimum level in the PS method, the model output is almost perfectly fitted to the target AP waveform, which indicates that the *I*_tot_cell_ in Eqs. (1) and (2) of two parameter sets are almost the same. If the two parameters *I*_X1sf_ and *I*_X2sf_ have ambiguity, these parameters in the final results of the PS method have multiple value combinations, however, they have almost identical *I*_tot_cell_. For example, if both *I*_X1sf_ and *I*_X2sf_ are the parameters of the inward current, then the increase in *I*_X1sf_ requires a decrease in *I*_X2sf_ to satisfy quasi-identical *I*_tot_cell_ (see Discussion in ^25^ for detail). To quantify this relationship, correlations were calculated for all 15 pairs of *I*_xsf_s in the top 20 MSE results of the multi-run PO method in TC12 cell where the two waveforms indicated by the leftmost and the middle red traces in Fig. 3C were used in two-waveform fitting (AB), the leftmost red trace only (A), and the middle red trace only (B). The correlation coefficient (R^2^) was divided in four grades of (a) 1 > R^2^ > 0.8, (b) 0.8 > R^2^ > 0.6, and (c) 0.6 > R^2^ > 0.4 and 0.4 > R^2^ > 0. From the above consideration, we can expect that if the number of the correlation of grade (a) is small, then the problem has small ambiguities. In Fig. 6, the grade of R^2^ was represented by the thickness of the line, connecting each pair of *I*_xsf_s. It is evident that the pattern of the 15 correlations obtained by the simultaneous two-waveform fitting (AB in Fig. 6) is distinct from those of the single waveform fittings of the control AP (A) or the *I*_Kr_ blocked AP (B). Thus, it is clear that the PO method based on two-waveform fitting is provided with more information if compared to the single waveform fitting.

The mechanisms underlying the variation of the correlation pattern may be complicated. Only, the disappearance of the correlation between *I*_hasf_ and any others in panel B was attributed to *I*_ha_ = 0. This is because the MDP is depolarized by the *I*_Kr_ block to a voltage range, where *I*_ha_ is totally deactivated.

### Physiological ionic mechanisms underlying the optimized AP waveforms

So far, the application of the simultaneous two-waveform fitting of the PS method was examined at the methodological point if the ionic current profile expressed on the surface membrane was correctly determined by using the error function MSE of the PO method independently of the initial parameter set. It should also be asked if the profile of current expression levels obtained by the PO method well matches the biophysical function of the cell, which is initiated by the membrane excitation relying on the physiological characteristics of individual currents.

**Figure 7:**
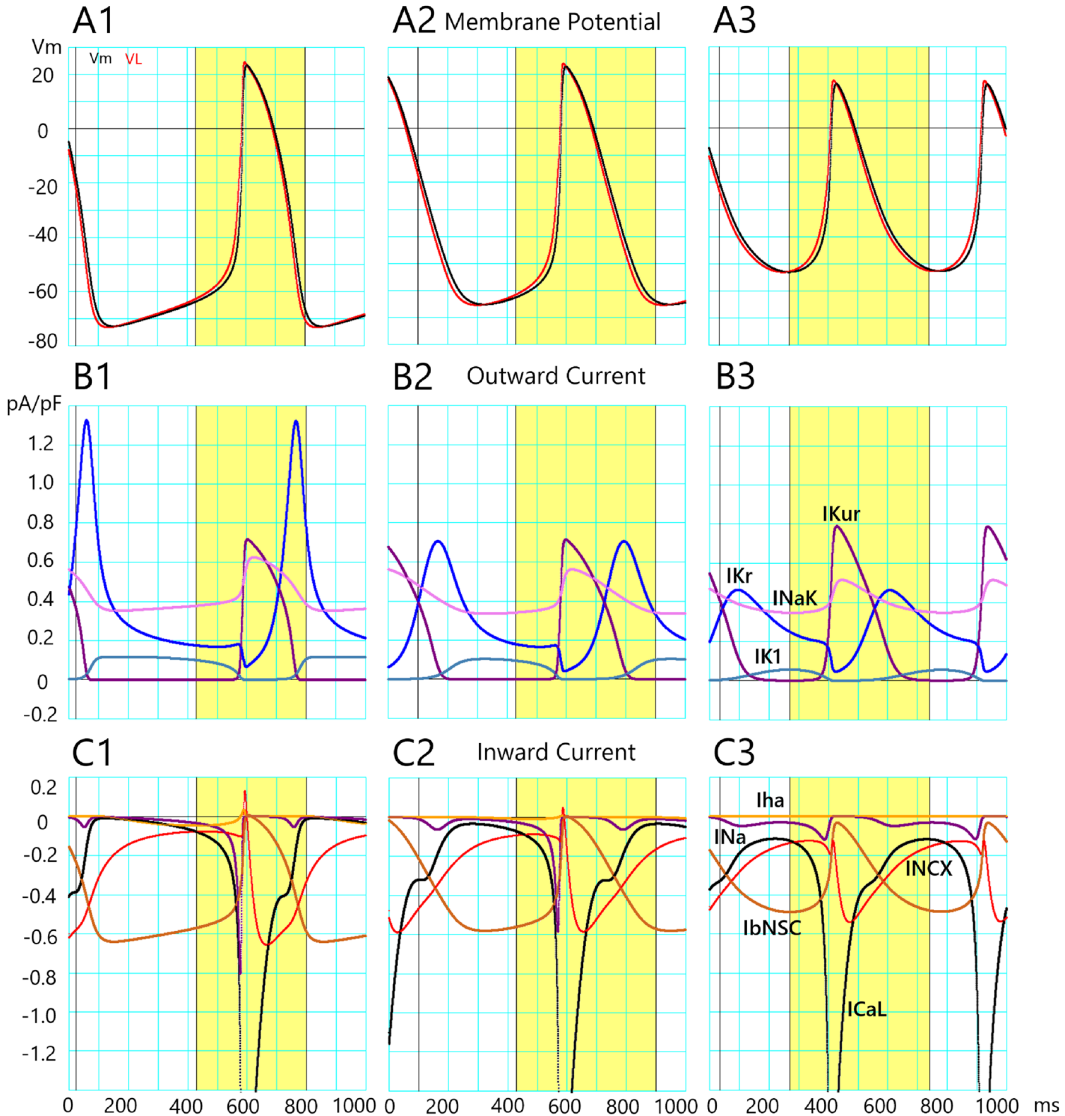
The profile of the current flow underlying the AP waveform of the cell-specific model of TC12 optimized by the PS method. The upper row (**A**) indicates V_m_ (black) and V_L_ (red). A1 indicates the control condition (c in Fig. 3C), and A2, A3 indicate the record obtained at two different degrees of the I_Kr_ block (b1 and b2 in Fig. 3C). The lower rows (**B**) and (**C**) indicate outward and inward I_m_, respectively. The individual I_m_ are depicted in different colors. The time scales indicated at the bottom are applied to both V_m_ and I_m_ panels. The yellow colors are used to highlight the mechanisms mainly for both the foot and plateau phases of AP. The peak of inward I_CaL_ is far beyond the graphic display.

**Figure 8:**
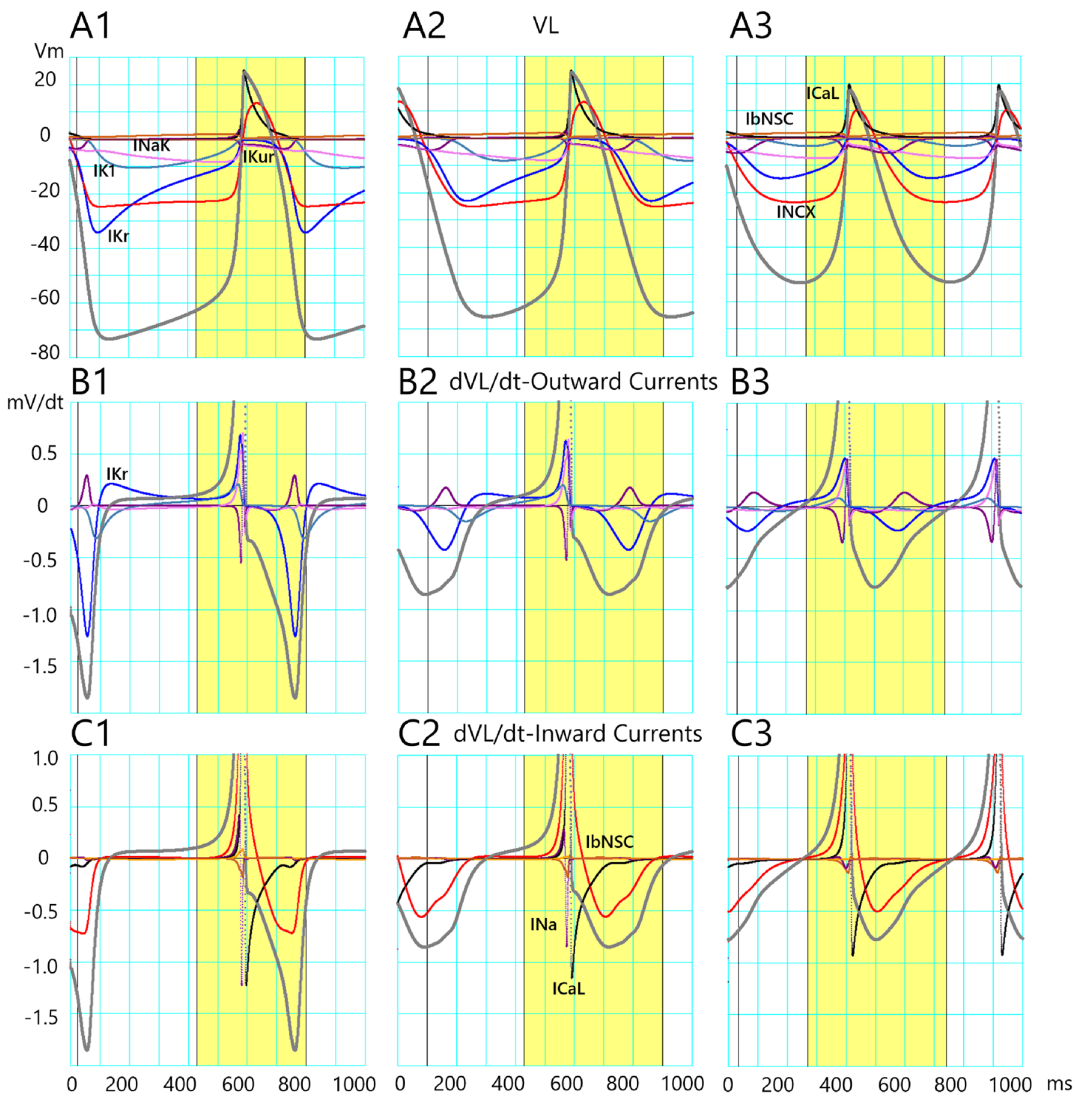
The profile of V_L_ and dV_L_/dt elements of the cell-specific model of TC12 revealed by the V_L_ analysis. The upper row (**A**) indicates V_L_ elements. A1 indicates the control condition (c in Fig. 3C), and A2, A3 indicate the record obtained at two different degrees of the I_Kr_ block. The lower rows (**B**) and (**C**) indicate dV_L_/dt elements for outward and inward currents, respectively. The individual V_L_ and dV_L_/dt elements are depicted in different colors. The time scales indicated at the bottom are applied to both V_L_ and dV_L_/dt records. The yellow colors are used to highlight the mechanisms underlying both the foot and plateau phases of AP. See the text for more explanation.

As demonstrated in Fig. 7A, the *V*_m_ (black trace) closely followed the time course of *V*_L_ (Eqs. 15 and 16); *V*_L_ > *V*_m_ during the depolarizing phase and vice versa (*V*_L_ < *V*_m_) during the repolarizing phase. The *V*_L_ crossed *V*_m_ at d*V*_m_/dt = 0 at OS and MDP. Since the time course of *V*_L_ was quite close to that of *V*_m_ (Fig. 7A), here the contribution of each current to *V*_m_ is described based on the corresponding changes in elements of both *V*_L_ and d*V*_L_/dt in Fig. 8.

Figure 7B,C shows the time course of individual membrane currents underlying the AP waveform (*V*_m_). In the control waveform in column 1 of Fig. 7 which corresponds to c in Fig. 3C, the *I*_Kr_ (blue) rapidly showed a large peak at about − 50 mV and then slowly decayed during SDD. The time course of *I*_NaK_ (pink) reflected its *V*_m_-dependency and the amplitude was relatively large if compared with others. *I*_K1_ (steel blue) was nearly zero during the plateau but showed a significant amplitude during SDD. The *I*_Kur_ (purple) was activated only during AP and provided the largest current during AP. As to the inward currents, *I*_bNSC_ (light brown) was the largest current during SDD and its amplitude was proportional to *V*_m_ because of the absence of time-dependent gating kinetics in *I*_bNSC_. The *I*_NCX_ current (red) transiently peaked in the outward direction at the foot of the AP upstroke, and the time course of the inward component reflected the Ca^2+^ transient; the inward peak appeared ~ 50 ms after the rising phase of AP, and thereafter it decayed. During the late half of SDD, the amplitude of both *I*_Na_ (purple) and *I*_CaL_ (black) increased exponentially due to the subthreshold *V*_m_-dependent activation. *I*_CaL_ was the major inward current during the plateau phase. In columns 2 and 3 of Fig. 7, *I*_Kr_ decreased gradually from control in column 1. Although *I*_xsf_s of all current species other than *I*_Kr_ were the same in three conditions in columns 1–3, the individual amplitude of each current in panels B and C varied according to their own kinetics when *V*_m_ changed as the result of *I*_Kr_ block.

The merit of the *V*_L_ analysis is its quantitative assessment of the contribution of individual membrane currents in mV, or mV/ms unit (in the case of *V*_L_, or d*V*_L_/dt, respectively) to the AP waveform (Fig. 7). Indeed, the *V*_L_ (gray) shown in Fig. 8A as well as in Fig. 7A (red) consists of the arithmetic sum of individual *V*_L_ elements (Eq. 15). Major *V*_L_ elements were *I*_NCX_, *I*_Kr_, *I*_K1_, *I*_NaK_ and *I*_bNSC_ during SDD, and *I*_CaL_, *I*_NCX_, and *I*_NaK_ during AP plateau. It should be noted that the time courses of individual *V*_L_ elements were all unique and quite different from those of the membrane currents, which provided a vital clue in exploring the direction of the PO by the PS method.

The time courses of individual elements of *V*_L_ were determined by the changes in the channel open probability as well as by the difference in (*V*_m_ − *E*_x_) in the case of the channel current. The time course of *I*_NaK_ and *I*_NCX_ elements are dependent not only on *V*_m_ but also largely determined by the [Na^+^]_i_ and [Ca^2+^]_i_. The Ca^2+^-induced Ca^2+^-release (CICR) is initiated by the Ca^2+^ influx through the LCC in the dyadic junction space. Then, the Ca^2+^-release channel on the SR membrane is activated by the massive increase in Ca^2+^ in the dyadic junction. The time course of this CICR is clearly visualized in the d*V*_L_/dt records (Fig. 8B,C). Namely, the onset of *I*_CaL_ activation is indicated by the rapid transient deflection of *I*_CaL_ element of d*V*_L_/dt which coincides with the rise of *I*_CaL_ element of *V*_L_. Then the transient upward deflection of the *I*_NCX_ element of d*V*_L_/dt indicates that the driving force (reversal potential of *I*_NCX_ − *V*_m_) is reversed during the overshoot of AP. Then the relatively slow downward deflection of the *I*_NCX_ element is driven by the increase of [Ca^2+^]_i_ within the dyadic space. This sequence of events was evident in the control (column 1 of Fig. 8) as well as in the two cases of *I*_Kr_-block (columns 2 and 3 of Fig. 8). The duration of SDD was shortened by both the delayed repolarization and the accelerated subthreshold activation of both *I*_Na_ and *I*_CaL_ because of the positive shift of MDP, both of which were caused by the *I*_Kr_-block (columns 2 and 3 of Fig. 8). With a more extensive *I*_Kr_ block (column 3 of Fig. 8), the SDD virtually disappeared because of the overlap between the delayed repolarization on the subthreshold activation of *I*_CaL_ and *I*_Na_.

These findings are totally consistent with the physiological mechanisms supposed so far through the experimental findings^1–4,10,40^.

## Discussion

### Brief summary of results

1. The parameter optimization method has been developed to determine the ionic current composition underlying the cardiac AP waveform. However, the method has not been established to be used in a wide range of electrophysiological research. It has been suggested that information included in a single AP waveform is still not enough for the PO method. In the present study, the AP record under the selective *I*_Kr_ block was used in addition to the control AP waveform.
2. The MM test of the simultaneous fitting of two AP waveforms was applied to the AP waveform modified by blocking one of the currents, *I*_Kr_, *I*_CaL_, *I*_Na_, *I*_Ks_, *I*_Kur_, or *I*_bNSC_ (Figs. 1 and 2, MM mode). In all cases of ion current block, the PS method successfully identified the blocked current, and the MSE was decreased fairly below log(MSE) < − 2.
3. In the EM test, the experimental record of the spontaneous AP frequently showed intermittent slow *V*_m_ fluctuations, which interfered with the gradient-based PO method. However, by using the multi-run test, the distribution of the *I*_xsf_ converged to a single point, even though the limit level of MSE decrease was largely enlarged in the EM test of the PS method (Fig. 4). The new method well resolved the *I*_Kr_-selective block current.
4. The correlations between two *I*_xsf_s were determined in 15 pairs of *I*_xsf_s. The pattern of 15 correlations was quite different among the three results obtained in the two-waveform fitting, and the single waveform fitting to the control and the *I*_Kr_ block recordings (Fig. 6). This finding supports the view that the simultaneous fitting method is provided with more information than the single AP waveform fitting, and resolves the unique current pattern underlying the membrane excitation in the cell-specific computer model.
5. The current flows of the major 7 currents in the optimized cell-specific model were analyzed by the *V*_L_ analysis method (Fig. 8). All these quantitative findings on the contributions of all currents were totally consistent with the qualitative ionic mechanisms of cardiac APs so far described in physiological literature. It is concluded that the parameter sets obtained by the present PS analysis are totally relevant to the physiological mechanisms underlying the AP waveform.

The importance of providing some information is required in addition to the single AP waveform when the PO method was applied to the cardiac AP. This point of improving the PO analysis has been extensively discussed in the literatures (see Kernik et al.^41^, Paci et al.^42^, Paci et al.^43^).

### The simultaneous fitting of two AP waveforms in the PS method.

The error function MSE is calculated from the difference between the target and model output AP waveforms to drive the PO. The waveform is determined by both the limiting conductance ${\overline{G} }_{x}$ and time-dependent changes in *pO* as described by Eq. (3). The *pO* is a critical factor in shaping the precise AP waveform according to the detailed *V*_m_-dependent rate constant for the channel gating (Eqs. 4 and 5). Particularly, the *V*_m_-dependency is quite different between different channels, so individual channel species can play distinct roles in different phases of APs. The spontaneous AP discharge provides an additional clue to the PO method in comparison to the fitting of the AP waveform triggered by the electrical stimulus in the conventional AP recordings. Namely, the profile of the multiple current flows during the SDD is totally different from that during the single AP, which is triggered by the electrical stimulation.

According to the above view, we used variable weights to calculate the weighted sum of MSE^25^. The largest weight (5 or 6) was applied to the MSE in the first segment of the falling phase after the plateau, where the *I*_Kr_ provided the dominant current. Namely, the *I*_Kr_ plays a key role in shaping the SDD as well as the APD in Figs. 7 and 8. The MSE of the second segment, SDD, was calculated using a weight of 1, and the last segment of the AP waveform with a weight of 0.2–0.5 since the current profile was largely determined during phases 1 and 2.

The amplitude of *I*_Na/K_ is largely determined by the [Na^+^]_i_. To avoid large fluctuations in [Na^+^]_i_, the activity of Na/K pump was regulated by an additional equation (Eqs. 12 and 13). Simultaneously, the [Ca]_t_ was also regulated by using the additional equation to regulate the *I*_NCX_. Thus, as indicated in Table 1 the final concentrations of these ions were quite stable within individual myocytes. It should be noted that the Ca^2+^ transient is dependent on the cellular content of Ca, which is composed of a total of free Ca^2+^ and the bound form Ca to various Ca buffer molecules.

### The slow *V*_m_ fluctuations in the record of spontaneous AP waveforms in hiPSC-CMs; the complication in the EM mode of the PO method.

The hiPSC-CMs show irregular slow fluctuations, which were difficult to reconstruct using exponential functions of the channel open-close kinetics (Eqs. 4 and 5). The slow fluctuations shifted the lower limit of MSE in the upward direction. Even under the condition of this extra noise, the multi-run test showed an evident peak of *I*_xsf_ demarcated by obvious slopes on both sides of the peak (Fig. 4). The minimum level of MSE was ~ 4 mV^2^, which was comparable to 3.29 ± 1.84 mV^2^ obtained in the single AP waveform method in our previous study^25^. This may be true if the MSE caused by slow fluctuations is virtually constant.

On the other hand, the slow *V*_m_ fluctuations should interfere with the progress of the PO. In the PS method, the BP is moved by ± step size, and the NP is moved toward smaller MSEs by the step size given by the PS method. By this maneuver, the *I*_xsf_ data points are located within a limited space in the multi-dimensional parameter space. The intermittent slow fluctuations in *V*_m_ may seriously interfere with the decision of the movement of NP (*I*_xsf_). Thereby, the NP will get out across the demarcation of the parameter space for a given *I*_xsf_ distribution to interrupt the PO at a modest MSE level. Although this mechanism may not explain all cases of interruption, the peak search using the multi-run test should largely compensate for the difficulty caused by the sporadic *V*_m_ fluctuations.

If the slow fluctuations of *V*_m_ are caused by an intervention of attaching the patch pipette to a fragile single hiPSC-CMs, the difficulty will be largely relieved by using a small cluster of a few numbers of hiPSC-CMs, provided that the cells are well coupled through the gap junction. Indeed, Doss et al.^38^ described that multiple impalements from each beating cluster yielded action potentials with similar morphology, suggesting that each cluster works as a functional syncytium. In the case of using matured cardiac ventricular myocytes, which are much larger compared to hiPSC-CMs, the slow fluctuations are not obvious under the physiological condition.

### Limitation of the present study; some discrepancy of the model from the intact myocytes

When the AP waveform was selected much later from the start of the *I*_Kr_ block in the experimental recordings, the fitting of the waveform showed obvious deviation to give larger MSEs (Fig. 3D). This was not observed in the MM fitting (Table 1). Thus, it may be suggested that the slow inactivation kinetics of *I*_CaL_ and/or *I*_NaL_ does not correctly represent the intact inactivation of these currents.

Alternatively, it may be suggested that model equations in the hVC model might be largely renewed in reference to recent publications in the near future.

### The merit of the simultaneous two-AP-waveform fitting in the PS method.

The PO can be conducted if the multiple PO parameters are independent of each other. For example, in the simple case of enzyme reaction, the rate of increase of product concentration, the rate of product concentration (*V*_p_) is a function of the three parameters, *V*_max_, *K*_0.5_, and n in Eq. (23).

23 $${V}_{p}=\frac{{V}_{max}}{1+\frac{{K}_{0.5}}{{S}^{n}}}$$

These three parameters are independent of each other. This means that changing the maximum rate of the reaction, *V*_max_ during the application of the PS method alters only the maximum rate of the enzyme reaction, but the half maximum substrate concentration (*K*_0.5_) as well as the Hill coefficient n are not affected. The same is true when *K*_0.5_ or n were adjusted. However, if *K*_0.5_ is replaced by the three rate constants for binding (*α*) and unbinding (*β*) of the substrate (*S*) to the enzyme (*E*), and production of the product (P) (*γ*) in the reaction, *α* and *β* do not converge in the multi-run test using the dose–response curve.

$$E + n \cdot S\begin{array}{*{20}c} {\mathop{\longrightarrow}\limits^{\alpha }} \\ {\xleftarrow[\beta ]{}} \\ \end{array} ES_{n} \mathop{\longrightarrow}\limits^{\gamma }n \cdot P + E$$

But the final distribution of scaling factors of *sf*_*α*_ and *sf*_*β*_ at the end of the multi-run test perfectly fit the liner correlation equation (Eq. 24) with the two constants of *α* and *β*.

24 $${sf}_{\alpha }=a\times {sf}_{\beta }+b$$

Indeed, in the study of Kohjitani et al.^25^, it was demonstrated that the dispersion of the parameters was much improved by fixing one of the parameters included in the co-relationship. In the simultaneous fitting to two waveforms in the PS method, variable co-relations will be added. If so, the final distribution of parameters might be limited within a smaller space in the multi-dimensional space.

### The convergence of *I*_xsf_s in multi-run test

The convergence of *I*_xsf_ is a critical index to judge the correctness of the estimation of *I*_xsf_. Also, the gradient of MSE/delta *I*_xsf_ could be a critical index of the sensitivity of the model to *I*_xsf_. However, unfortunately, the *I*_xsf_ is rather arbitrarily determined in the present study. For comparison of the distribution between different currents, it might be necessary to normalize the magnitude of *I*_xsf_ by adjusting *I*_xmag_. Alternatively, the $\Delta MSE/\Delta {I}_{\text{xsf}}$ which is calculated during the process of the PS method might be used to determine the sensitivity of the model to *I*_xsf_. Also, the uniqueness of *I*_xsf_s is important in the PO method, while it is generally difficult to theoretically prove it. In our previous report^25^ using single-waveform optimization, the uniqueness was shown in Fig. 1 by the stochastic method, where the results strongly supported the single minimum of the problem. In this two-waveform optimization, S.D. values in Table 1 may support the uniqueness of the minimum, where S.D. values were calculated from the top 20 results of the multi-run test.

### Limitation of the parameter optimization method

The PO method used in this work is a gradient based method, which has limitations. The gradient based optimization methods usually require good initial values to obtain accurate results, thus in this work, we first adjusted all parameters manually. The PO methods also have limitations in the number of parameters to be optimized. In this work, we found that the maximum number of the optimization parameters is six, thus in the manual adjustment process, we examined which parameters are effective in forming the target AP waveforms and selected maximum six parameters to be optimized. However, as described in the method, in principle, there is no theoretical limit to the number of parameters that can be optimized, where it can be up to the number of model parameters, as long as the parameters in the model do not interfere with each other or are independent of each other. These limitations may be improved by using a more sophisticated algorithm such as hybrid optimization methods.

### Supplementary Information

Supplementary Information.

## Data Availability

Computer programs used for the two-AP waveform fitting and VL analysis in Results and that for the PS method applied to a simple enzyme reaction model in Discussion are coded in Visual Basic. The computer programs and the datasets used and analyzed during the current study are available from the corresponding author upon reasonable request.

## List of symbols

Parameters: (Channel; Gene)
*I*_*Na*_: (Na_V_ 1.5; *SCN5A*)
*I*_*CaL*_: (Ca_V_1.2; *CACNA1*)
*I*_*CaT*_: (Ca_V_ 3.1; *CACNA1G*)
*I*_ha_: (HCN4)
*I*_*st*_: (Ca_V_1.3; *CACNA1D*)
*I*_*bNSC*_: (Not identified)
*I*_*K1*_: (Kir2.1; *KCNJ2*)
*I*_*Kr*_: (K_V_11.1; *KCNH2*)
*I*_*Ks*_: (K_V_7.1; *KCNQ1/KCNE1*)
*I*_*Kto*_: (K_V_4.3; *KCND3*)
*I*_*Kur*_: (K_V_1.5; *KCNA5*)
*I*_*NCX*_: (NCX1; *SLC8A1*)
*I*_*NaK*_: (Na^+^-K^+^ ATPase; *ATP1A1*)

## Abbreviations

APD: Action potential duration
BP: Base point for searching minimum MSE in the PS
EM test/mode: An experimental AP waveform is compared with the model output of a computer model in the PO method
hiPSC-CM: Human induced pluripotent stem cell-derived cardiomyocytes
hVC model: The human ventricular cell model
MDP: Maximum diastolic potential
MM test/mode: The output of a computer model is compared with the output of the same model after randomization of the parameters in the PO method
MSE: Mean square difference in two AP waveforms
NP: Searching point in reference to BP in the PS
OS: Overshoot potential of AP
PO method: Parameter optimization method, which adjusts one or more model parameters to fit with the target data
PS method: Pattern search method, which is one of the gradient-based parameter optimization methods
SDD: Slow diastolic depolarization
