# Supplementary material for: Cell-specific models of hiPSC-CMs developed by the gradient-based parameter optimization method fitting two different action potential waveforms
Source: Sci Rep. 2024 Jun 7;14:13086. doi: 10.1038/s41598-024-63413-0 (PMC11161598; doi:10.1038/s41598-024-63413-0)
Supplement: Supplementary file 1 — Supplementary Information. [file 41598_2024_63413_MOESM1_ESM.pdf]

Supplemental Materials

Cell-specific models of hiPSC-CMs developed by the gradient-based parameter optimization method fitting two different action potential waveforms

Authors: Yixin Zhang, Futoshi Toyoda, Yukiko Himeno*, Akinori Noma, Akira Amano

The source code of the computer model is available from ResearchGate or e-Heart website.

Abbreviations

Table S1. Abbreviations in model equations

V_m	membrane potential (mV)
I_{tot_cell}	total current of ion channels and exchangers (pA/pF)
$I_{tot\_x\_a}$	total current of ion 'x' channels and exchangers at space 'a' (pA/pF)
I_{app}	current applied through a patch electrode (pA/pF)
$E_{rev,x}$	reversal potential of current 'x' (mV) determined from the tangential line of the I_x - V curve
C_m	whole cell membrane capacitance (pF)
G_x	conductance of current 'x' (pA /pF/mV)
k, α, β	rate constants (ms^{-1} or $mM^{-1}ms^{-1}$)
$P_{I(\_X)}$	converting factor of GHK_X from mM/ms to pA/mM/pF
ν_{cyc_x}	turnover rate of transporter 'x' (ms^{-1})
V_a	total volume of space 'a' (fL)
$[X_{total}]_a$	total concentration of substance 'X' at space 'a' (mM)
$[X_{free}]_a$	concentration of free substance 'X' at space 'a' (mM)
$[X]_a$	concentration of 'X' at space 'a' (mM)
J_X	total flux of ion 'X' (attomol/ms)
$\frac{d[X]_a}{dt}$	rate of change of 'X' concentration at space 'a' (mM/ms)

Model Parameters

Table S2. Physical constants

R	8.3143	C·mV/mmol/K
T	310.15	K
F	96.4867	C/mmol

Table S3. Ion concentrations

$[K^+]_o$	5.4	mM
$[Na^+]_o$	145	mM
$[Ca^{2+}]_o$	1.8	mM

GHK equation

The magnitudes of ion channel currents are described either by the Ohmic equation or by the GHK equation. In the latter case, the term to convert mM to pA (permeability times, *zF*) in the original GHK equation is represented by a lumped converting factor, P in a unit of pA/pF mM^{-1}, because of unknown total number of channels within a cell and single channel conductance. Then, the amplitude of current (I) for an ion X is given by,

$$I = P \cdot pO \cdot GHK_X \qquad Eq.S1$$

Where GHK_X is,

$$GHK_X = \frac{Z_X^2 F^2 V_m}{RT} \cdot \frac{\left([X]_i - [X]_o \cdot exp\left(\frac{-Z_X F V_m}{RT}\right)\right)}{\left(1 - exp\left(\frac{-Z_X F V_m}{RT}\right)\right)} \qquad Eq.S2$$

Nernst equation

$$E_X = \frac{RT}{z_X F} \cdot ln\left(\frac{[X]_o}{[X]_i}\right) \qquad Eq.S3$$

Table S4. Volume composition of cytosol in comparison with HuVEC model [1,2]

	hiPSC-CM	HuVEC model
Input capacitance	32 pF	192.46 pF
Cell volume (V_{cell})	2510 fL	37920 fL
Bulk space (V_{blk})	65% of V_{cell} fL	68% of V_{cell} fL
Intermediate zone (V_{iz})	3.5% of V_{cell} fL	3.5% of V_{cell} fL
Junctional space (V_{jnc})	0.8% of V_{cell} fL	0.8% of V_{cell} fL
Total SR space (V_{SR})	1.1% of V_{cell} = 27.61 fL	6% of V_{cell} fL
Volume of SR releasing site (V_{SRrl})	10% of V_{SR} fL	20% of V_{SR} fL
Volume of SR releasing site (V_{SRup})	90% of V_{SR} fL	80% of V_{SR} fL

Ca^{2+} buffer

The detailed set of buffer species used in the GPB model [3] was adopted after several simplifications as described in our previous paper [1]. In short, we deleted the myosin, Na^+ and Mg^{2+} buffers, and fixed [Mg^{2+}]. The low affinity binding of Ca^{2+} to troponin (TnCl) was replaced by a contraction model [4] and the amount of the high affinity site (TnCh) was adjusted.

Bulk space (blk)

$$\frac{d[CaMCa]}{dt} = k_{on\_CaM} \cdot [Ca^{2+}]_{blk} \cdot ([B_{total}CaM] - [CaMCa]) - k_{off\_CaM} \cdot [CaMCa] \quad Eq.S4$$

$$k_{off\_CaM} = 0.0238, k_{on\_CaM} = 3.4, [B_{total}CaM] = 0.024 \quad Eq.S5$$

$$\frac{d[TnChCa]}{dt} = k_{on\_TnCh} \cdot [Ca^{2+}]_{blk} \cdot ([B_{total}TnCh] - [TnChCa]) - k_{off\_TnCh} \cdot [TnChCa] \quad Eq.S6$$

$$k_{off\_TnCh} = 0.000032, k_{on\_TnCh} = 2.37, [B_{total}TnCh] = 0.007 \quad Eq.S7$$

$$\frac{d[SRCa]}{dt} = k_{on\_SR} \cdot [Ca^{2+}]_{blk} \cdot ([B_{total}SR] - [SRCa]) - k_{off\_SR} \cdot [SRCa] \quad Eq.S8$$

$$k_{off\_SR} = 0.006, k_{on\_SR} = 10, [B_{total}SR] = 0.0171 \quad Eq.S9$$

Intermediate zone (iz)

$$[L_{free}]_{iz} = \frac{[B_{total}L]_{iz}}{1 + \frac{[Ca^{2+}]_{iz}}{K_{dL\_iz}}}, [B_{total}L] = 0.6078 \qquad Eq.S10$$

$$K_{dL\_iz} = \frac{k_{off\_L\_iz}}{k_{on\_L\_iz}}, k_{off\_L\_iz} = 1.3, k_{on\_L\_iz} = 100 \qquad Eq.S11$$

$$[H_{free}]_{iz} = \frac{[B_{total}H]_{iz}}{1 + \frac{[Ca^{2+}]_{iz}}{K_{dH\_iz}}}, [B_{total}H] = 0.2178 \qquad Eq.S12$$

$$K_{dH\_iz} = \frac{k_{off\_H\_iz}}{k_{on\_H\_iz}}, k_{off\_H\_iz} = 0.03, k_{on\_H\_iz} = 100 \qquad Eq.S13$$

$$[Ca^{2+}]_{iz} = \frac{[Ca_{total}]_{iz}}{1 + \frac{[Lf]_{iz}}{K_{dL\_iz}} + \frac{[Hf]_{iz}}{K_{dH\_iz}}} \qquad Eq.S14$$

Junctional space (jnc)

$$[L_{free}]_{jnc} = \frac{[B_{total}L]_{jnc}}{1 + \frac{[Ca^{2+}]_{jnc}}{K_{dL\_jnc}}}, [B_{total}L] = 1.1095 \qquad Eq.S15$$

$$K_{dL\_jnc} = \frac{k_{off\_L\_jnc}}{k_{on\_L\_jnc}}, k_{off\_L\_jnc} = 1.3, k_{on\_L\_jnc} = 100 \qquad Eq.S16$$

$$[H_{free}]_{jnc} = \frac{[B_{total}H]_{jnc}}{1 + \frac{[Ca^{2+}]_{jnc}}{K_{dH\_jnc}}}, [B_{total}H] = 0.398 \qquad Eq.S17$$

$$K_{dH\_jnc} = \frac{k_{off\_H\_jnc}}{k_{on\_H\_jnc}}, k_{off\_H\_jnc} = 0.03, k_{on\_H\_jnc} = 100 \qquad Eq.S18$$

$$[Ca^{2+}]_{jnc} = \frac{[Ca_{total}]_{jnc}}{1 + \frac{[Lf]_{jnc}}{K_{dL\_jnc}} + \frac{[Hf]_{jnc}}{K_{dH\_jnc}}} \qquad Eq.S19$$

Release site of the SR (SRrl)

$$k_{off\_CSQN} = 65, k_{on\_CSQN} = 100, [B_{total}CSQN] = 10 \qquad Eq.S20$$

$$K_{d\_CSQN\_Ca} = \frac{k_{off\_CSQN}}{k_{on\_CSQN}} \qquad Eq.S21$$

$$a = 1 \qquad Eq.S22$$

$$b = [B_{total}CSQN] - [Ca^{2+}_{total}]_{SRrl} + K_{d\_CSQN\_Ca} \qquad Eq.S23$$

$$c = -K_{d\_CSQN\_Ca} \cdot [Ca_{total}]_{SRrl} \qquad Eq.S24$$

$$[Ca^{2+}]_{SRrl} = \frac{-b + \sqrt{b^2 - 4ac}}{2a} \qquad Eq.S25$$

Boundary Ca^{2+} diffusion

Ca^{2+} transfer between cytosolic compartments

$$J_{Ca\_jnciz} = G_{dCa\_jnciz} \cdot \left([Ca^{2+}]_{jnc} - [Ca^{2+}]_{iz}\right) \qquad Eq.S26$$

$$G_{dCa\_jnciz} = 32158\ (fL \cdot ms^{-1}) \qquad Eq.S27$$

$$J_{Ca\_izblk} = G_{dCa\_izblk} \cdot ([Ca^{2+}]_{iz} - [Ca^{2+}]_{blk}) \qquad Eq.S28$$

$$G_{dCa\_izblk} = 2076.1\ (fL \cdot ms^{-1}) \qquad Eq.S29$$

Ca2+ transfer from SR uptake site to release site

$$J_{trans\_SR} = P_{trans} \cdot \left([Ca^{2+}]_{SRup} - [Ca^{2+}]_{SRrl}\right) \qquad Eq.S30$$

$$P_{trans} = 0.017(fL \cdot ms^{-1}) \qquad Eq.S31$$

Ion channels and transporters

L-type Ca^{2+} current (I_{CaL}, LCC)

According to the scheme of Shirokov et al. [5] and Ferreira et al. [6], the same 4-state model was used for both LCCs in CaRU (I_{CaL_jnc}) and for LCCs located in *blk* (I_{CaL_blk}) and *iz* (I_{CaL_iz}). The description of both V_m-dependent gate and $[Ca^{2+}]$-dependent gates in HuVEC model was used in the hiPSC-CM model after minor modification as in Kohjitani et al. [7] model. The rate constants for the V_m-gate (α_+ and α_-) and Ca^{2+}-gate (ε_+ and ε_-) of LCC are given by *Eqs. S35, S36* and *Eqs. S37, S38*, respectively. Both activation (α_+) and deactivation (α_-) rates of the V_m-gate were described as a function of two exponential terms and adjusted to hiPSC-CM data.

$$I_{CaL\_X\_a} = f_{CaL\_a} \cdot P_{CaL\_X} \cdot GHK_{X\_a} \cdot pO_{LCC\_a} \cdot \frac{1}{1 + \left(\frac{1.4}{[ATP]}\right)^3} \cdot us\_CaL \qquad Eq.S32$$

[ATP] was fixed to 6 mM.

Fraction of I_{CaL}

$$f_{CaL\_jnc} = 0.15,\ f_{CaL\_blk} = 0.45,\ f_{CaL\_iz} = 0.40 \qquad Eq.S33$$

Converting factors

$$P_{CaL\_Ca} = 5.068,\ P_{CaL\_Na} = 0.0000185 \cdot P_{CaL\_Ca},\ P_{CaL\_K} = 0.000367 \cdot P_{CaL\_Ca}\ (pA/pF/mM)$$

The rate constants for the V_m-gate,

$$v = V_m - V_{shiftCa} \qquad \text{Eq.}S34$$

$$\alpha_+ = \frac{1}{0.763 \cdot exp\left(-\frac{v}{8.5}\right) + 0.348 \cdot exp\left(-\frac{v}{3500}\right)} \qquad Eq.S35$$

$$\alpha_{-} = \frac{0.5}{4.65 \cdot exp\left(\frac{v}{15}\right) + 1.363 \cdot exp\left(\frac{v}{100}\right)} \qquad Eq.S36$$

The rate constant (ε_{+}) for the Ca^{2+}-inactivation.

$$\varepsilon_{+} = \frac{0.35 \cdot [Ca^{2+}]_{nd} \cdot \alpha_{+}}{T_L \cdot K_L} \qquad Eq.S37$$

The values of T_L (= 147.51) and K_L (=0.00396 mM) were determined by referring to the experimental measurements of steady-state inactivation. The rate of removing Ca^{2+} inactivation (ε_{-}) used in HuVEC model was used.

$$\varepsilon_{-} = \frac{1}{8084 \cdot exp\left(\frac{V_m}{10}\right) + 158 \cdot exp\left(\frac{V_m}{1000}\right)} + \frac{1}{134736 \cdot exp\left(-\frac{V_m}{5}\right) + 337 \cdot exp\left(-\frac{V_m}{2000}\right)} \qquad Eq.S38$$

The ultraslow gate was described by the 2-state model. The rate constants for the ultraslow gate, the forward rate (K_{slowf}) and the backward rate (K_{slowb}) were indicated below.

$$v = V_m - V_{shiftus} \qquad Eq.S39$$

$$K_{slowf\_CaL} = \frac{0.55}{5000000 \cdot exp\left(\frac{v}{6}\right) + 100 \cdot exp\left(\frac{v}{65}\right)} \qquad Eq.S40$$

$$K_{slowb\_CaL} = \frac{0.55}{1000 \cdot exp\left(-\frac{v}{14}\right) + 66 \cdot exp\left(-\frac{v}{65}\right)} \qquad Eq.S41$$

The composition of whole cell I_{CaL}.

$$I_{CaL} = \left(I_{CaL\_Ca\_jnc} + I_{CaL\_Na\_jnc} + I_{CaL\_K\_jnc}\right) + \left(I_{CaL\_Ca\_iz} + I_{CaL\_Na\_iz} + I_{CaL\_K\_iz}\right) + \left(I_{CaL\_Ca\_blk} + I_{CaL\_Na\_blk} + I_{CaL\_K\_blk}\right) \qquad Eq.S42$$

The sustained inward current (*Ist*)

In the spontaneous SA node cells, a sustained inward current was activated on depolarization to more negative potential range (V_m =~ -60 mV) than the usual threshold of the L-type Ca^{2+} current. The characteristics of the current was roughly similar to I_{CaL}, except that I_{st} was resistant to the removal of Ca^{2+} from the external solution and it was suggested that I_{st} is most probably carried by Na^{+} [8,9]. Recently, Toyoda et al. [10,11] suggested that this current is generated by Cav.1.3, which is the major subtype expressed in the SA node cells. In the present study, we calculated I_{st} in the iPSC_CMs for convenience of comparing the role of I_{st} between the iPSC_CMs and the matured SA node cells. If appropriate, the sum of (I_{st} and the conventional I_{CaL}) was calculated.

$$I_{st} = I_{st,Na} + I_{st,K} \qquad \text{Eq.}S43$$

$$I_{st,Na} = P_{stNa} \cdot GHK_{Na} \cdot pO \quad P_{stNa} = 0.00236\, pA/pF/mM \qquad \text{Eq.}S44$$

$$I_{st,K} = P_{stK} \cdot GHK_{K} \cdot pO \quad P_{stK} = 0.585 \cdot P_{stNa}\, pA/pF/mM \qquad \text{Eq.}S45$$

$$P_O = d \cdot f \cdot u \qquad \text{Eq.}S46$$

$$(1-y) \underset{\beta}{\overset{\alpha}{\rightleftarrows}} y \qquad y = \{d, f, u\} \qquad \text{Eq.}S47$$

$$\alpha_d = \frac{1}{0.15 \cdot Exp(\frac{V_m}{-11}) + 0.2 \cdot Exp(\frac{V_m}{-700})} \qquad \text{Eq.}S48$$

$$\beta_d = \frac{1}{16 \cdot Exp(\frac{V_m}{8}) + 15 \cdot Exp(\frac{V_m}{50})} \qquad \text{Eq.}S49$$

$$\alpha_f = \frac{1}{3100 \cdot Exp(\frac{V_m}{13}) + 700 \cdot Exp(\frac{V_m}{70})} \qquad \text{Eq.}S50$$

$$\beta_f = \frac{1}{95 \cdot Exp(\frac{V_m}{-10}) + 50 \cdot Exp(\frac{V_m}{-700})} + \frac{2.5 \cdot [Ca^{2+}]_{blk}}{1 + Exp(\frac{V_m}{-5})} \quad \text{Eq.}S51$$

$$\alpha_u = \frac{1}{400000 \cdot Exp(\frac{V_m}{9}) + 60 \cdot Exp(\frac{V_m}{65})} \quad \text{Eq.}S52$$

$$\beta_u = \frac{1}{700 \cdot Exp(\frac{V_m}{-14}) + 60 \cdot Exp(\frac{V_m}{-65})} \quad \text{Eq.}S53$$

T-type Ca^{2+} current (I_{CaT})

I_{CaT} is assumed in the *blk* space.

$$I_{CaT} = 2 \cdot P_{CaT} \cdot GHK_{Ca} \cdot pO_{CaT},\ P_{CaT} = 9.56 \quad Eq.S54$$

$$pO_{CaT} = d \cdot f \quad Eq.S55$$

$$\alpha_d = \frac{1}{0.019 \cdot exp\left(-\frac{V_m}{5.6}\right) + 0.82 \cdot exp\left(-\frac{V_m}{250}\right)} \quad Eq.S56$$

$$\beta_d = \frac{1}{40 \cdot exp\left(\frac{V_m}{6.3}\right) + 1.5 \cdot exp\left(\frac{V_m}{10000}\right)} \quad Eq.S57$$

$$\alpha_f = \frac{1}{62000 \cdot exp\left(\frac{V_m}{10.1}\right) + 30 \cdot exp\left(\frac{V_m}{3000}\right)} \quad Eq.S58$$

$$\beta_f = \frac{1}{0.0006 \cdot exp\left(-\frac{V_m}{6.7}\right) + 1.2 \cdot exp\left(-\frac{V_m}{25}\right)} \quad Eq.S59$$

The hyperpolarization-activated current (I_{ha} or I_f)

In 1976, Noma and Irisawa [12] for the first time conducted the double-microelectrode voltage clamp in a man-made small tissue preparation (0.2~0.3 mm in diameter) of the rabbit SA node tissue. They found a very slow activation time course of inward current (I_h) on

hyperpolarization from the holding potential of -40 mV. Yanagihara and Irisawa [13] clearly separated I_h from the delayed rectifier K current by the difference in the activation range and the Ba^{2+}-resistant nature of I_{ha}. They measured the fully activated I-V relationship with the reversal potential is at -25 mV, suggesting little sensitivity to any particular ion species. They developed the Hodgkin-Huxley type kinetic model of I_{ha}, and suggested that I_{ha} plays a significant role in keeping the pacemaker cell at a low membrane potential, but only a small role in promoting the slow diastolic depolarization because of its time constant of several seconds. Yanagihara et al. [14] published the mathematical model of the SA node cell action potential. The detailed I_{ha} model described by Maruoka et al. [15] was used to reflect the delay in both activation and deactivation on hyper- and de-polarizations, respectively.

$$I_{ha} = I_{ha,Na} + I_{ha,K} \qquad \text{Eq.}S60$$

$$I_{ha,Na} = P_{ha,Na} \cdot GHK_{Na} \cdot pO \quad P_{ha,Na} = 0.03642 \quad pA/pF/mM \qquad \text{Eq.}S61$$

$$I_{ha,K} = P_{ha,K} \cdot GHK_K \cdot pO \quad P_{ha,K} = 4.244 \cdot P_{ha,Na} \quad pA/pF/mM \qquad \text{Eq.}S62$$

$$C1 \underset{\lambda}{\overset{\mu}{\rightleftarrows}} C2 \underset{\beta}{\overset{\alpha}{\rightleftarrows}} O1 \underset{\beta}{\overset{\alpha}{\rightleftarrows}} O2 \underset{\beta}{\overset{\alpha}{\rightleftarrows}} O3 \qquad \text{Eq.}S63$$

$$\alpha_{ha} = \frac{1}{3500 \cdot \exp(\frac{V_m}{16.8}) + 0.3 \cdot \exp(\frac{V_m}{400})} \qquad \text{Eq.}S64$$

$$\beta_{ha} = \frac{1}{4 \cdot \exp(\frac{V_m}{-14}) + 2 \cdot \exp(\frac{V_m}{-400})} \qquad \text{Eq.}S65$$

$$\mu_{ha} = \frac{1}{45000000 \cdot \exp(\frac{V_m}{8.7}) + 500 \cdot \exp(\frac{V_m}{200})} \qquad \text{Eq.}S66$$

$$\lambda_{ha} = \frac{1}{10.5 \cdot \exp(\frac{V_m}{-16.4}) + 0.4 \cdot \exp(\frac{V_m}{-400})}$$

Eq. $S67$

In the steady-state, the full model is reduced to a two-state transition model of a closed state (Ct) and an open state (Ot) to obtain the steady-state open probability (pOt$_{SS}$)

$$Ct \underset{kb}{\overset{kf}{\rightleftarrows}} Ot \qquad \text{Eq.}S68$$

where,

$$kf = \frac{\alpha}{\frac{\lambda}{\mu}+1} \qquad kb = \frac{\beta}{1+\frac{\alpha}{\beta}+(\frac{\alpha}{\beta})^2} \qquad pOt_{SS} = \frac{kf}{kf+kb} \qquad \text{Eq.}S69$$

Sodium current (*I_{Na}*)

The same I_{Na} model as in our previous study [1] was used, except for the amplitude parameters, f_L and P_{Na}. I_{Na} is composed of the two components, I_{NaT} and I_{NaL}. The scheme for the state transition is shown below.

$$I_{Na} = I_{NaT} + I_{NaL} \qquad Eq.S70$$

$$f_L = \frac{I_{NaL}}{I_{NaT}+I_{NaL}} = 0.04\ or\ 0.01 \qquad Eq.S71$$

$$I_{NaT} = (1 - f_L) \cdot P_{Na} \cdot (GHK_{Na} + 0.18 \cdot GHK_K) \cdot p(O)_{NaT} \qquad Eq.S72$$

$$I_{NaL} = f_L \cdot P_{Na} \cdot (GHK_{Na} + 0.18 \cdot GHK_K) \cdot p(O)_{NaL} \qquad Eq.S73$$

$$P_{Na\_Na} = 73.77825, P_{Na\_K} = 0.18 \cdot P_{Na_{Na}}\ (pA/pF/mM)$$

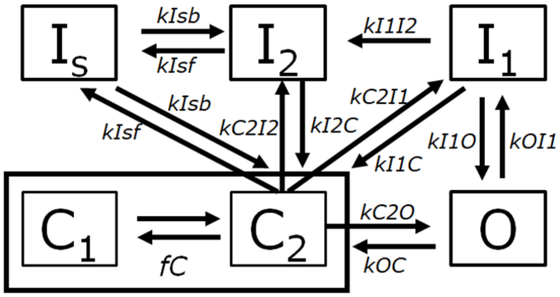

Transient component (I_{NaT})

$$\frac{dp(O)_{NaT}}{dt} = k_{I_2O} \cdot p(I_2)_{NaT} + f_{C\_Na} \cdot k_{C_2O} \cdot p(C)_{NaT} - \left(k_{OC} + k_{OI_2}\right) \cdot p(O)_{NaT} \qquad Eq.S74$$

$$\frac{dp(I_2)_{NaT}}{dt} = f_{C\_Na} \cdot k_{C_2I_2} \cdot p(C)_{NaT} + k_{OI_2} \cdot p(O)_{NaT} + k_{I_sb} \cdot p(I_s)_{NaT} - \left(k_{I_2C} + k_{I_2O} + k_{I_sf}\right) \cdot p(I_2)_{NaT} \qquad Eq.S75$$

$$\frac{dp(I_s)_{NaT}}{dt} = k_{I_sf} \cdot p(I_2)_{NaT} + k_{I_sf} \cdot p(C)_{NaT} - 2 \cdot k_{I_sb} \cdot p(I_s)_{NaT} \qquad Eq.S76$$

$$p(C)_{NaT} = 1.0 - p(I_s)_{NaT} - p(O)_{NaT} - p(I_2)_{NaT} \qquad Eq.S77$$

$$f_{C\_Na} = \frac{C_2}{C_1 + C_2} = \frac{1}{1 + exp\left(-\frac{v+48}{7}\right)} \qquad Eq.S78$$

$$v = Vm - VshiftNa \qquad \text{Eq. S79}$$

$$k_{C_2O} = \frac{1}{0.0025 \cdot exp\left(-\frac{v}{8.0}\right) + 0.15 \cdot exp\left(-\frac{v}{100.0}\right)} \qquad Eq.S80$$

$$k_{OC} = \frac{1}{30.0 \cdot exp\left(\frac{v}{12.0}\right) + 0.53 \cdot exp\left(\frac{v}{50.0}\right)} \qquad Eq.S81$$

$$k_{OI_2} = \frac{1}{0.0433 \cdot exp\left(-\frac{V_m}{27.0}\right) + 0.34 \cdot exp\left(-\frac{V_m}{2000.0}\right)} \qquad Eq.S82$$

$$k_{I_2O} = 0.0001312 \qquad Eq.S83$$

$$k_{C_2I_2} = \frac{0.5}{1.0 + \frac{k_{I_2O} \cdot k_{OC}}{k_{OI_2} \cdot k_{C_2O}}} \qquad Eq.S84$$

$$k_{I_sb} = \frac{1}{300000.0 \cdot exp\left(\frac{V_m}{10.0}\right) + 50000.0 \cdot exp\left(\frac{V_m}{16.0}\right)} \quad Eq.S85$$

$$k_{I_sf} = \frac{1}{0.016 \cdot exp\left(-\frac{V_m}{9.9}\right) + 8.0 \cdot exp\left(-\frac{V_m}{45.0}\right)} \quad Eq.S86$$

Late component (*I_{NaL}*)

The $k_{I_1I_2}$, k_{OI_1}, k_{I_1O}, k_{I_1C} and $k_{C_2I_1}$ are specific for I_{NaL}, and other rate constants are the same as in I_{NaT}.

$$\frac{dp(O)_{NaL}}{dt} = k_{I_1O} \cdot p(I_1)_{NaL} + f_{C\_Na} \cdot k_{C_2O} \cdot p(C)_{NaL} - \left(k_{OC} + k_{OI_1}\right) \cdot p(O)_{NaL} \quad Eq.S87$$

$$\frac{dp(I_1)_{NaL}}{dt} = f_{C\_Na} \cdot k_{C_2I_1} \cdot p(C)_{NaL} + k_{OI_1} \cdot p(O)_{NaL} - \left(k_{I_1C} + k_{I_1O} + k_{I_1I_2}\right) \cdot p(I_1)_{NaL} \quad Eq.S88$$

$$\frac{dp(I_2)_{NaL}}{dt} = f_{C\_Na} \cdot k_{C_2I_2} \cdot p(C)_{NaL} + k_{I_1I_2} \cdot p(I_1)_{NaL} + k_{I_sb} \cdot p(I_s)_{NaL} - \left(k_{I_2C} + k_{I_sf}\right) \cdot p(I_2)_{NaL} \quad Eq.S89$$

$$\frac{dp(I_s)_{NaL}}{dt} = k_{I_sf} \cdot p(I_2)_{NaL} + k_{I_sf} \cdot p(C)_{NaL} - 2 \cdot k_{I_sb} \cdot p(I_s)_{NaL} \quad Eq.S90$$

$$p(C)_{NaL} = 1.0 - p(I_s)_{NaL} - p(O)_{NaL} - p(I_1)_{NaL} - p(I_2)_{NaL} \quad Eq.S91$$

$$k_{I_1I_2} = 0.00534 \quad Eq.S92$$

$$k_{OI_1} = k_{OI_2} \quad Eq.S93$$

$$k_{I_1O} = 0.01 \quad Eq.S94$$

$$k_{I_1C} = k_{I_2C} \quad Eq.S95$$

$$k_{C_2I_1} = k_{C_2I_2} \quad Eq.S96$$

Inward rectifier potassium current (*I_{K1}*)

The I_{K1} model developed by Yan and Ishihara [16] and Ishihara and Yan [17] was used in HuVEC model [2] after modifying several parameters.

$$I_{K1} = G_{K1} \cdot (V_m - E_K) \cdot p(O)_{K1} \qquad Eq.S97$$

$$G_{K1} = \frac{0.451773 \cdot \left(\frac{[K^+]_O}{5.4}\right)^{0.4}}{1 + exp\left(-\frac{[K^+]_O - 2.2}{0.6}\right)} \, nS/pF \qquad Eq.S98$$

$$p(O)_{K1} = pO_{mode1} + pO_{mode2} \qquad Eq.S99$$

Mode 1: the channel block by Mg^{2+} and spermine (SPM)

The SPM-block is a time-dependent process, while the Mg^{2+}-block is instantaneous.

$$Pbspm \underset{\beta \cdot pOMg}{\overset{\alpha}{\rightleftarrows}} 1 - Pbspm \qquad \text{Eq.}S100$$

$$\alpha_{Mg} = 12.0 \cdot exp\left(-\frac{V_m - E_K}{40}\right) \qquad Eq.S101$$

$$\beta_{Mg} = 28.0 \cdot exp\left(-\frac{V_m - E_K}{40}\right) \cdot [Mg^{2+}]_{cyt} \qquad Eq.S102$$

$$f_O = \frac{\alpha_{Mg}}{\alpha_{Mg} + \beta_{Mg}} \qquad Eq.S103$$

$$f_B = \frac{\beta_{Mg}}{\alpha_{Mg} + \beta_{Mg}} \qquad Eq.S104$$

$$pO_{Mg} = f_O \cdot f_O \cdot f_O \qquad Eq.S105$$

$$pO_{Mg1} = 3.0 \cdot f_O \cdot f_O \cdot f_B \qquad Eq.S106$$

$$pO_{Mg2} = 3.0 \cdot f_O \cdot f_B \cdot f_B \qquad Eq.S107$$

$$pB_{Mg3} = f_B \cdot f_B \cdot f_B \qquad Eq.S108$$

$$\alpha_{SPM} = \frac{0.17 \cdot exp\left(-0.07 \cdot \left((V_m - E_K) + 8 \cdot [Mg^{2+}]_{cyt}\right)\right)}{1.0 + 0.01 \cdot exp\left(0.12 \cdot \left((V_m - E_K) + 8 \cdot [Mg^{2+}]_{cyt}\right)\right)} \qquad Eq.S109$$

$$\beta_{SPM} = \frac{0.28 \cdot [SPM] \cdot exp\left(0.15 \cdot \left((V_m - E_K) + 8 \cdot [Mg^{2+}]_{cyt}\right)\right)}{1.0 + 0.01 \cdot exp\left(0.13 \cdot \left((V_m - E_K) + 8 \cdot [Mg^{2+}]_{cyt}\right)\right)} \qquad Eq.S110$$

$$\frac{dPb_{SPM}}{dt} = \beta_{SPM} \cdot pO_{Mg} \cdot (1 - Pb_{SPM}) - \alpha_{SPM} \cdot Pb_{SPM} \qquad Eq.S111$$

$$pO_{mode1} = 0.9 \cdot (1 - Pb_{SPM}) \cdot \left(pO_{Mg} + \frac{2}{3}pO_{Mg1} + \frac{1}{3}pO_{Mg2}\right) \qquad Eq.S112$$

Mode 2: the channel block only by SPM

The channel is free from the Mg-block, and the SPM is instantaneous.

$$Pbspm \underset{Kd}{\longleftrightarrow} [SPM] \cdot (1 - Pbspm)$$

$$pO_{mode2} = \frac{0.1}{1+\frac{[SPM]}{Kd}} \quad Kd = 40 \cdot exp\left(-\frac{V_m - E_K}{9.1}\right) mM \qquad Eq.S113$$

Delayed rectifier K$^+$ current, fast component (I_{Kr})

We installed the I_{Kr} model developed by Ono and Ito [18], which well fitted the result of experimental I_{Kr} data of hiPSC-CMs [19].

The current amplitude is described with an Ohmic equation.

$$I_{Kr} = G_{Kr} \cdot (V_m - E_K) \cdot p(O)_{Kr} \qquad Eq.S114$$

$$G_{Kr} = 0.049644 \cdot \left(\frac{[K^+]_O}{5.4}\right)^{0.2} nS/pF \qquad Eq.S115$$

The open probability of the channel is described with three gating parameters, y_1, y_2, and y_3, each of which is calculated by a two-state transition scheme.

$$p(O)_{Kr} = (0.6 \cdot y_1 + 0.4 \cdot y_2) \cdot y_3 \qquad Eq.S116$$

$$\frac{dy_N}{dt} = \alpha_{y_N} \cdot (1.0 - y_N) - \beta_{y_N} \cdot y_N, \qquad N = 1, 2, 3 \qquad Eq.S117$$

$$\alpha_{y_1} = \frac{1}{20 \cdot exp\left(-\frac{V_m + 6}{6}\right) + 5 \cdot exp\left(-\frac{V_m + 6}{150}\right)} \qquad Eq.S118$$

$$\beta_{y_1} = \frac{1}{160 \cdot exp\left(\frac{(V_m + 6)}{28}\right) + 200 \cdot exp\left(\frac{(V_m + 6)}{1000}\right)} + \frac{1}{2500 \cdot exp\left(\frac{(Vm + 6)}{20}\right)} \qquad Eq.S119$$

$$\alpha_{y_2} = \frac{1}{200 \cdot exp\left(-\frac{(V_m + 6)}{6.5}\right) + 20 \cdot exp\left(-\frac{(V_m + 6)}{150}\right)} \qquad Eq.S120$$

$$\beta_{y_2} = \frac{1}{1600 \cdot exp\left(\frac{(V_m + 6)}{28}\right) + 2000 \cdot exp\left(\frac{(V_m + 6)}{1000}\right)} + \frac{1}{10000 \cdot exp\left(\frac{(Vm + 6)}{20}\right)} \qquad Eq.S121$$

$$\alpha_{y_3} = \frac{1}{10 \cdot exp\left(\frac{(V_m + 6)}{17}\right) + 2.5 \cdot exp\left(\frac{(V_m + 6)}{300}\right)} \qquad Eq.S122$$

$$\beta_{y_3} = \frac{1}{0.35 \cdot exp\left(-\frac{(V_m + 6)}{17}\right) + exp\left(-\frac{(V_m + 6)}{75}\right)} \qquad Eq.S123$$

Delayed rectifier K$^+$ current, slow component (*I_{Ks}*)

$$I_{Ks\_K} = P_{Ks} \cdot GHK_K \cdot p(O)_{Ks}\,,\ P_{Ks} = 0.4\ (pA/pF/mM) \qquad Eq.S124$$

$$I_{Ks\_Na} = 0.04 \cdot P_{Ks} \cdot GHK_{Na} \cdot p(O)_{Ks} \qquad Eq.S125$$

$$p(O)_{Ks} = (O_v)^2 \cdot (0.99 \cdot O_C + 0.01) \qquad Eq.S126$$

The V$_m$-dependent gate

$$\alpha_{v_{Ks}} = \frac{1}{150 \cdot exp\left(-\frac{(V_m + 10)}{25}\right) + 900 \cdot exp\left(-\frac{(V_m + 10)}{200}\right)} \qquad Eq.S127$$

$$\beta_{v_{Ks}} = \frac{1}{1000 \cdot exp\left(\frac{(V_m + 10)}{13}\right) + 220 \cdot exp\left(\frac{(V_m + 10)}{50}\right)} \qquad Eq.S128$$

$$\frac{dO_v}{dt} = \alpha_{v_{Ks}} \cdot (1.0 - O_v) - \beta_{v_{Ks}} \cdot O_v \qquad Eq.S129$$

The Ca^{2+}-dependent gate

$$C1 \underset{\beta 3}{\overset{\alpha 3}{\rightleftarrows}} C2 \underset{\beta 2}{\overset{\alpha 2}{\rightleftarrows}} Oc$$

$$\frac{dO_c}{dt} = -\alpha_2 \cdot C_2 - \beta_2 \cdot O_c \qquad \text{Eq. } S130$$

$$\frac{dC_2}{dt} = \alpha_3 \cdot C_1 - \beta_3 \cdot C_2 - \alpha_2 \cdot C_2 + \beta_2 \cdot O_c \qquad \text{Eq. } S131$$

$$C_1 = 1.0 - C_2 - O_c \qquad \text{Eq. } S132$$

$$\alpha_3 = 0.0003, \qquad \beta_3 = 0.03 \qquad Eq.S133$$

$$\alpha_2 = 2.25 \cdot [Ca^{2+}], \qquad \beta_2 = 0.000296 \qquad Eq.S134$$

Transient outward K+ current (I_{Kto})

$$I_{Kto\_K} = P_{Kto} \cdot GHK_K \cdot p(O)_{Kto}\,, P_{Kto} = 0.01729\ (pA/pF/mM) \qquad Eq.S135$$

$$I_{Kto\_Na} = 0.09 \cdot P_{Kto} \cdot GHK_{Na} \cdot p(O)_{Kto} \qquad Eq.S136$$

$$p(O)_{Kto} = y_{1_{Kto}} \cdot y_{2_{Kto}} \qquad Eq.S137$$

$$\frac{dy_{1_{Kto}}}{dt} = \alpha_{y_1} \cdot \left(1.0 - y_{1_{Kto}}\right) - \beta_{y_1} \cdot y_{1_{Kto}}, \quad \frac{dy_{2_{Kto}}}{dt} = \alpha_{y_2} \cdot \left(1.0 - y_{2_{Kto}}\right) - \beta_{y_2} \cdot y_{2_{Kto}} \qquad Eq.S138$$

$$\alpha_{y_1} = \frac{1}{13 \cdot exp\left(-\frac{V_m}{22}\right)}, \qquad \beta_{y_1} = \frac{1}{2.1 \cdot exp\left(\frac{V_m}{90}\right)} \qquad Eq.S139$$

$$\alpha_{y_2} = \frac{0.5}{950 \cdot exp\left(\frac{V_m}{500}\right)}, \qquad \beta_{y_2} = \frac{0.5}{40 \cdot exp\left(-\frac{V_m}{9}\right) + 13 \cdot exp\left(-\frac{V_m}{1000}\right)} \qquad Eq.S140$$

Ultra-rapid K+ current (I_{Kur})

I_{Kur} model in a mouse ventricular cell model [20] is used.

$$I_{Kur} = G_{Kur} \cdot (a_{ur})^3 \cdot i_{ur} \cdot (V_m - E_K) \qquad Eq.S141$$

$$G_{Kur} = 0.000113 \cdot \left(1 + \frac{1}{1 + exp\left(-\frac{V_m - 30}{59}\right)}\right) nS/pF \qquad Eq.S140$$

$$\frac{da_{ur}}{dt} = \alpha_{ur} \cdot (1.0 - a_{ur}) - \beta_{ua} \cdot a_{ur}, \qquad \frac{di_{ur}}{dt} = \alpha_{ui} \cdot (1.0 - i_{ur}) - \beta_{ui} \cdot i_{ur} \qquad Eq.S143$$

$$\alpha_{ua} = \frac{1}{0.65 \cdot exp\left(-\frac{(V_m + 19)}{8.5}\right) + exp\left(-\frac{(V_m - 21)}{59}\right)}, \qquad \beta_{ua} = \frac{1}{0.65 \cdot \left(2.5 + exp\left(\frac{(V_m + 91)}{17}\right)\right)} \quad Eq.S144$$

$$\alpha_{ui} = \frac{1}{21 + exp\left(-\frac{(V_m - 185)}{28}\right)}, \qquad \beta_{ui} = exp\left(\frac{V_m - 158}{16}\right) \qquad Eq.S145$$

Time-independent currents

All these currents are from Takeuchi et al. [21] as described in Asakura et al. [1].

Background Ca^{2+} current (I_{bCa})

$$I_{bCa\_a} = P_{bCa\_a} \cdot 2 \cdot GHK_{Ca}, \qquad a = (blk, iz) \qquad Eq.S146$$

$$P_{bCa} = 0.00125 \ \ (pA/pF/mM) \qquad Eq.S147$$

Background non-selective cation current (I_{bNSC})

$$I_{bNSC\_X} = P_{bNSC_X} \cdot GHK_X, \qquad X = (K, Na) \qquad Eq.S148$$

$$P_{bNSC\_Na} = 0.000182875, P_{bNSC\_K} = 0.4 \cdot P_{bNSC\_Na}(pA/pF/mM) \qquad Eq.S149$$

$$I_{bNSC} = I_{bNSC\_K} + I_{bNSC\_Na} \qquad Eq.S150$$

Calcium-activated background cation current ($I_{l(Ca)}$)

$$p(O)_a = \frac{1.0}{1.0 + \left(\frac{0.0012}{[Ca^{2+}]_a}\right)^3} \qquad Eq.S151$$

$$I_{l(Ca)\_X\_a} = P_{l(Ca)\_X\_a} \cdot f_{l(Ca)\_X\_a} \cdot GHK_X \cdot p(O)_a, \qquad X = (Na, K), \qquad a = (blk, iz) \quad Eq.S152$$

$$P_{l(Ca)\_Na} = 0.01375 \ (pA/pF/mM) \qquad Eq.S153$$

$$P_{l(Ca)\_K} = P_{l(Ca)\_Na} \ (pA/pF/mM) \qquad Eq.S154$$

Fraction of $I_{l(Ca)}$

$$f_{l(Ca)\_iz} = 0.1, f_{l(Ca)\_blk} = 0.9 \qquad Eq.S155$$

$$I_{l(Ca)} = I_{l(Ca)\_Na\_iz} + I_{l(Ca)\_K\_iz} + I_{l(Ca)\_Na\_blk} + I_{l(Ca)\_K\_blk} \qquad Eq.S156$$

ATP-sensitive potassium current (I_{KATP})

$$p(O)_{KATP} = \frac{1}{1.0 + \left(\frac{[ATP]_{cyt}}{0.1}\right)^2} \quad Eq.S157$$

$$\chi_{KATP} = 0.0236 \cdot ([K^+]_o)^{0.24} \quad Eq.S158$$

$$I_{KATP} = G_{KATP} \cdot (V_m - E_K) \cdot p(O)_{KATP} \cdot \chi_{KATP} \quad Eq.S159$$

$$G_{KATP} = 18.75 \quad Eq.S160$$

Na^+/K^+ pump current (I_{NaK})

The Na^+/K^+ pump model developed by Oka et al. [22] on the framework of Smith and Crampin [23] was used after adjusting the amplitude as indicated in the main text in Eqs. 12 and 13.

Na^+/Ca^{2+} exchange current (I_{NCX})

The NCX model developed by Takeuchi et al. [21] was used after adjusting the amplitude similarly as in I_{NaK}.

CaRU

The model of CaRU described in the HuVEC model was used. The model structure of CaRU is shown in Fig. S1. Derivation of the instantaneous $[Ca^{2+}]_{nd}$, which is sensed by both LCC and RyRs for inactivation and activation, respectively, was originally given in Hinch [24] and modified by Himeno et al. [2]. In short, the volume of *nd* was assumed to be virtually zero so that the $[Ca^{2+}]_{nd}$ can be given by the instantaneous equation Eq. S161,

$$[Ca^{2+}]_{nd} = \frac{[Ca^{2+}]_{jnc} + \frac{g_R}{g_d} \cdot [Ca^{2+}]_{SRrl} + \frac{g_L}{g_d} \cdot \frac{\delta V \cdot e^{-\delta V}}{1 - e^{-\delta V}} \cdot [Ca^{2+}]_o}{(1 + \frac{g_R}{g_d} + \frac{g_L}{g_d} \cdot \frac{\delta V \cdot e^{-\delta V}}{1 - e^{-\delta V}})} \qquad \text{Eq. } S161$$

where Ca^{2+} fluxes through LCC and RyR, and diffusion from *nd* to *jnc* (J_L, J_R and J_D) were given as Eqs. S162-164.

$$J_L = g_L \cdot \frac{\delta V \cdot e^{-\delta V}}{1 - e^{-\delta V}} \cdot ([Ca^{2+}]_o - [Ca^{2+}]_{nd}) \qquad \text{Eq. } S162$$

$$J_R = g_R \cdot ([Ca^{2+}]_{SRrl} - [Ca^{2+}]_{nd}) \qquad \text{Eq. } S163$$

$$J_D = g_D \cdot ([Ca^{2+}]_{nd} - [Ca^{2+}]_{jnc}) \qquad \text{Eq. } S164$$

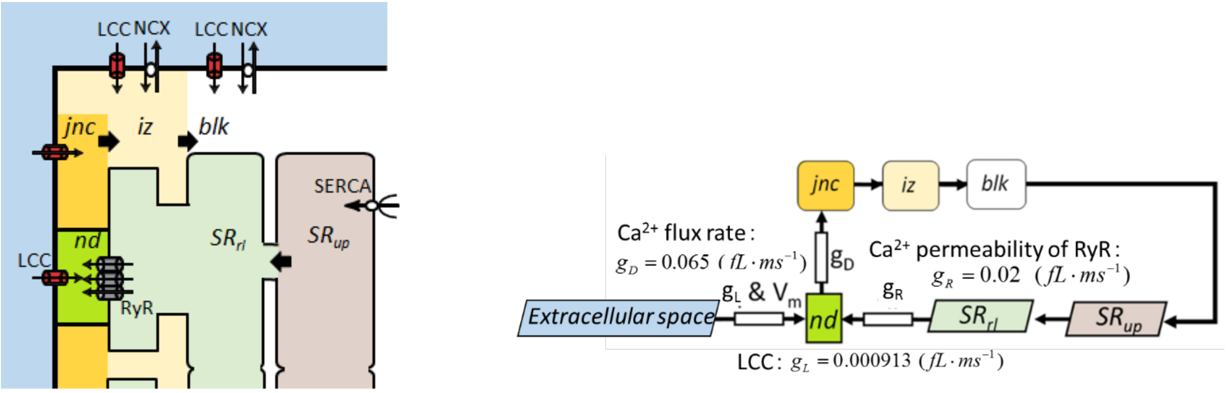

Fig. S1 Model structure of CaRU in relation to three Ca^{2+} diffusion compartments (left) and direction of Ca^{2+}diffusion (right) within the cell model. The L-type Ca^{2+} channel (LCC) and NCX are located on the sarcolemma, SERCA and RyRs are on the SR membrane. A single CaRU consists of a hypothetical LCC and a couplon (a cluster of RyRs) in the junctional cleft (filled with lime green), and individual CaRUs are spatially separated from its neighbors by jnc. The inset on the right shows schematic presentation of the diffusion pathway of Ca^{2+} from the Ca^{2+} sources (SR or extracellular space) to the sink (nd) and then to the cytoplasm (jnc, iz and blk). J_L, J_R and g_D represent permeability of single LCC and RyR, and Ca^{2+} flux rate from nd to jnc, respectively.

Sarcoplasmic reticulum Ca^{2+} pump (SERCA) current (J_{SERCA})

The three-state model developed by Tran et al. [25] was used after several minor modifications as described in Asakura et al. [1]. The limiting amplitude of J_{SERCA}, Amp_{SERCA}, was modified.

Rate of change in the membrane potential and ion concentrations

Membrane potential

$$\frac{dV_m}{dt} = -\left(I_{tot\_cell} + I_{app}\right) \qquad Eq.S165$$

$$I_{tot\_cell} = I_{tot\_Ca} + I_{tot\_Na} + I_{tot\_K} \qquad Eq.S166$$

$$I_{tot\_Ca} = I_{tot\_Ca\_jnc} + I_{tot\_Ca\_iz} + I_{tot\_Ca\_blk} \qquad Eq.S167$$

$$I_{tot\_Ca\_jnc} = I_{CaL\_Ca\_LR} + I_{CaL\_Ca\_L0} \qquad Eq.S168$$

$$I_{tot\_Ca\_iz} = I_{CaL\_Ca\_iz} + I_{NCX\_Ca\_iz} + I_{Cab\_iz} \qquad Eq.S169$$

$$I_{tot\_Ca\_blk} = I_{CaL\_Ca\_blk} + I_{CaT} + I_{Cab\_blk} + I_{NCX\_Ca\_blk} \qquad Eq.S170$$

$$I_{tot\_Na} = \left(I_{CaL\_Na\_jnc} + I_{CaL\_Na\_iz} + I_{CaL\_Na\_blk}\right) + \left(I_{NCX\_Na\_iz} + I_{NCX\_Na\_blk}\right) + \left(I_{Ks\_Na\_iz} + I_{Ks\_Na\_blk}\right) + I_{NaT\_Na} + I_{NaL\_Na} + I_{NaK\_Na} + I_{Kto\_Na} + I_{bNSC\_Na} + \left(I_{LCCa\_Na\_iz} + I_{LCCa\_Na\_blk}\right) + I_{st\_Na} + I_{ha\_Na} \qquad Eq.S171$$

$$I_{tot\_K} = \left(I_{CaL\_K\_jnc} + I_{CaL\_K\_iz} + I_{CaL\_K\_blk}\right) + I_{NaT\_K} + I_{NaL\_K} + I_{K1} + I_{Kur} + I_{Kpl} + I_{Kr} + \left(I_{Ks\_K\_iz} + I_{Ks\_K\_blk}\right) + I_{Kto\_K} + I_{KATP\_K\_cyt} + I_{bNSC\_K} + \left(I_{LCCa\_K\_iz} + I_{LCCa\_K\_blk}\right) + I_{NaK\_K} + I_{KACh} + I_{st\_K} + I_{ha\_K} \qquad Eq.S172$$

Ion concentrations

$$\frac{d[Ca_{total}]_{jnc}}{dt} = -\frac{I_{tot\_Ca\_jnc} \cdot C_m}{V_{jnc} \cdot 2 \cdot F} + \frac{J_{Ca\_rel}}{V_{jnc}} - \frac{J_{Ca\_jnciz}}{V_{jnc}} \qquad Eq.S173$$

$$\frac{d[Ca_{total}]_{iz}}{dt} = -\frac{I_{tot\_Ca\_iz} \cdot C_m}{V_{iz} \cdot 2 \cdot F} + \frac{J_{Ca\_jnciz}}{V_{iz}} - \frac{J_{Ca\_izblk}}{V_{iz}} \qquad Eq.S174$$

$$\frac{d[Ca_{total}]_{blk}}{dt} = -\frac{I_{tot\_Ca\_blk} \cdot C_m}{V_{blk} \cdot 2 \cdot F} + \frac{J_{Ca\_izblk}}{V_{blk}} - \frac{J_{Ca\_SERCA}}{V_{blk}} \qquad Eq.S175$$

$$\frac{d[Ca^{2+}]_{SRup}}{dt} = \frac{J_{Ca\_SERCA}}{V_{SRup}} - \frac{J_{trans\_SR}}{V_{SRup}} \qquad Eq.S176$$

$$\frac{d[Ca_{total}]_{SRrl}}{dt} = \frac{J_{trans\_SR}}{V_{SRrl}} - \frac{J_{rel\_SR}}{V_{SRrl}} \qquad Eq.S177$$

$$\frac{d[Na^+]_i}{dt} = -\frac{I_{tot\_Na} \cdot C_m}{V_{cyt} \cdot F} \qquad Eq.S178$$

$$\frac{d[K^+]_i}{dt} = -\frac{(I_{tot\_K} + I_{app}) \cdot C_m}{V_{cyt} \cdot F} \qquad Eq.S179$$

Contraction

The original model of Negroni and Lascano [4] was used. The magnitude of F_b is given in a unit of mN·mm^{-2}. The binding of Ca^{2+} to a troponin system (TS) having 3 Ca^{2+} binding sites (given in μM) was included in the equation of determining the concentration of free Ca^{2+} in the bulk compartment.

$$[Ca^{2+}]_{blk} = [Ca_{total}]_{blk} - \left([CaMCa] + [TnChCa] + [SRCa] + \frac{3 \cdot ([TSCa_3] + [TSCa_3^{\sim}] + [TSCa_3^{*}])}{1000}\right)$$

$$Eq.S180$$

References

1. Asakura, K., Cha, C. Y., Yamaoka, H., Horikawa, Y., Memida, H., Powell, T., et al. EAD and DAD mechanisms analyzed by developing a new human ventricular cell model. *Progress in Biophysics and Molecular Biology* 116, 11–24. (2014).

2. Himeno, Y., Asakura, K., Cha, C. Y., Memida, H., Powell, T., Amano, A., et al. A Human Ventricular Myocyte Model with a Refined Representation of Excitation-Contraction Coupling. *Biophysical Journal* 109, 415–427. (2015).

3. Grandi, E., Pasqualini, F. S., and Bers, D. M. A novel computational model of the human ventricular action potential and Ca transient. *J. Mol. Cell. Cardiol.* 48, 112–121. (2010).

4. Negroni, J. A., and Lascano, E. C. Simulation of steady state and transient cardiac muscle response experiments with a Huxley-based contraction model. *J. Mol. Cell. Cardiol.* 45, 300–312. (2008).

5. Shirokov, R., Levis, R., Shirokova, N., and Ríos, E. Ca(2+)-dependent inactivation of cardiac L-type Ca2+ channels does not affect their voltage sensor. *The Journal of General Physiology* 102, 1005–1030. (1993).

6. Ferreira, G., Yi, J., Ríos, E., and Shirokov, R. Ion-dependent inactivation of barium current through L-type calcium channels. *The Journal of General Physiology* 109, 449–461. (1997).

7. Kohjitani, H., Koda, S., Himeno, Y. et al. Gradient-based parameter optimization method to determine membrane ionic current composition in human induced pluripotent stem cell-derived cardiomyocytes. Sci Rep. 12, 19110 (2022).

8. Guo J, Ono K, Noma A. A sustained inward current activated at the diastolic potential range in rabbit sino-atrial node cells. *J. Physiol. (Lond.)* 483:1-13. (1995).

9. Mitsuiye T, Guo J, Noma A. Nicardipine-sensitive Na^+-mediated single channel currents in guinea-pig sinoatrial node pacemaker cells. *JPhysiol* (Lond) 521: 69-79. (1999).

10. Toyoda F, Mesirca P, Dubel S, Ding WG, Striessnig J, Mangoni ME, Matsuura H. Cav1.3 L-type Ca^{2+} channel contributes to the heartbeat by generating a dihydropyridine-sensitive persistent Na^+ current. Sci. Rep. Aug 11;7(1):7869. (2017).

11. Toyoda F, Ding WG, Matsuura H. Heterogeneous functional expression of the sustained inward Na + current in guinea pig sinoatrial node cells. *Pflugers Arch* 470(3), 481-490. (2018).

12. Noma A, Irisawa H. Membrane currents in the rabbit sinoatrial node cell as studied by the double microelectrode method. *Pflugers Arch*. 364(1):45–52. (1976).

13. Yanagihara K, Irisawa H. Inward current activated during hyperpolarization in the rabbit sinoatrial node cell. *Pflugers Arch* 385(1):11–19. (1980).

14. Yanagihara K, Noma A, Irisawa H. Reconstruction of sino-atrial node pacemaker potential based on the voltage clamp experiments. *Jpn J Physiol.* 30(6):841–857. (1980a).

15. Maruoka F, Nakashima Y, Takano M, Ono K, Noma A. Cation-dependent gating of the hyperpolarization-activated cation current in the rabbit sino-atrial node cells. *J Physiol* (Lond) 477, 423–435. (1994).

16. Yan, D.-H., and Ishihara, K. Two Kir2.1 channel populations with different sensitivities to Mg(2+) and polyamine block: a model for the cardiac strong inward rectifier K(+) channel. *J. Physiol. (Lond.)* 563, 725–744. (2005).

17. Ishihara K, Yan DH. Low-affinity spermine block mediating outward currents through Kir2.1 and Kir2.2 inward rectifier potassium channels. *J Physiol* 583(Pt 3), 891-908. (2007).

18. Ono, K., and Ito, H. Role of rapidly activating delayed rectifier K+ current in sinoatrial node pacemaker activity. *Am. J. Physiol.* 269, H453–62. (1995).

19. Ma, J., Guo, L., Fiene, S. J., Anson, B. D., Thomson, J. A., Kamp, T. J., et al. High purity human-induced pluripotent stem cell-derived cardiomyocytes: electrophysiological

properties of action potentials and ionic currents. *Am. J. Physiol. Heart Circ. Physiol.* 301, H2006–17. (2011).

20. Bondarenko, V. E., Szigeti, G. P., Bett, G. C. L., Kim, S.-J., and Rasmusson, R. L. Computer model of action potential of mouse ventricular myocytes. *American Journal of Physiology - Heart and Circulatory Physiology* 287, H1378–403. (2004).

21. Takeuchi, A., Tatsumi, S., Sarai, N., Terashima, K., Matsuoka, S., and Noma, A. Ionic mechanisms of cardiac cell swelling induced by blocking Na^+/K^+ pump as revealed by experiments and simulation. *The Journal of General Physiology* 128, 495–507. (2006).

22. Oka, C., Cha, C. Y., and Noma, A. Characterization of the cardiac Na+/K+ pump by development of a comprehensive and mechanistic model. *Journal of Theoretical Biology* 265, 68–77. (2010).

23. Smith, N. P., and Crampin, E. J. Development of models of active ion transport for whole-cell modelling: cardiac sodium-potassium pump as a case study. *Progress in Biophysics and Molecular Biology* 85, 387–405. (2004).

24. Hinch R. A mathematical analysis of the generation and termination of calcium sparks. *Biophys J* 86(3), 1293-307. (2004).

25. Tran, K., Smith, N. P., Loiselle, D. S., and Crampin, E. J. A thermodynamic model of the cardiac sarcoplasmic/endoplasmic Ca(2+) (SERCA) pump. *Biophysical Journal* 96, 2029–2042. (2009).
